# Targeted Ruthenium‐Based Anti‐Inflammatory Nanoagent for Enhanced Rheumatoid Arthritis Treatment

**DOI:** 10.1002/EXP.20240043

**Published:** 2025-08-16

**Authors:** Ziwei Zhao, Hao Xiong, Jinyong Wu, Shiyu Xu, Lihua Zhao, Yanshuai Wang, Shuai Chen, Cunyi Fan, Dechao Niu

**Affiliations:** ^1^ Lab of Low‐Dimensional Materials Chemistry, Key Laboratory for Ultrafine Materials of Ministry of Education, Frontier Science Center of the Materials Biology and Dynamic Chemistry, School of Materials Science and Engineering East China University of Science and Technology Shanghai China; ^2^ Department of Orthopedics Shanghai Sixth People's Hospital Affiliated to Shanghai Jiao Tong University School of Medicine Shanghai China

**Keywords:** anti‐inflammatory, organosilica, photothermal, rheumatoid arthritis, ruthenium

## Abstract

The inhibition of joint synovial inflammation, caused by poor oxygen (O_2_) supply and excessive reactive oxygen species (ROS) generation, is an important treatment strategy for rheumatoid arthritis (RA). Herein, we formulated a targeted ruthenium‐based anti‐inflammatory nanosystem consisting of ruthenium clusters‐loaded F127‐organosilica micelles with folic acid (FA) modification (RuFOMs‐FA) for RA treatment through a two‐stage macrophage regulatory mechanism. At the first stage, RuFOMs‐FA exhibited excellent photothermal capability with a high photothermal conversion efficiency of 55.3% upon external‐field 808 nm NIR irradiation, which further induced the death of M1 macrophages through the folic acid‐mediated active targeting pathway. Further, the resultant nanoagent mimicked enzymes displayed catalase‐like and superoxide dismutase‐like activities for endogenously scavenging ROS and producing O_2_ to induce the polarization of pro‐inflammatory M1 to anti‐inflammatory M2 macrophages in the RA physiological environment. More importantly, RuFOMs‐FA effectively alleviated hypoxia, inflammation, and cartilage destruction in the synovial joints in a rat RA model by the two‐stage macrophage regulatory mechanism. Consequently, it is highly expected that the developed RuFOMs‐FA could be applied as a new noble metal‐based anti‐inflammatory candidate nanosystem for efficient and safe RA treatment.

## Graphical abstract

A targeted ruthenium‐based anti‐inflammatory nanosystem (RuFOMs‐FA) based of ultra‐small ruthenium clusters‐loaded F127‐organosilica micelles with folic acid modification is designed and developed.

A two‐stage macrophage regulatory mechanism involving NIR external field‐induced M1 macrophage death and catalase/superoxide dismutase‐like activities‐mediated polarization of pro‐inflammatory M1 to anti‐inflammatory M2 macrophages through the folic acid‐mediated active targeting pathway, promising a new insight for efficient and safe rheumatoid arthritis treatment.

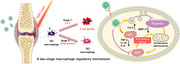

## 1 Introduction

Rheumatoid arthritis (RA) is an autoimmune disorder characterized by cartilage destruction and chronic synovitis and causing severe pain and joint disability in the affected patients [1, 2, 3, 4, 5]. Although the precise causes of RA are still unclear, it is well known that RA is an autoimmune condition that mainly affects the synovial joints [6, 7]. Macrophages play an important role in the pathophysiology of RA [8, 9, 10]. Macrophages can be polarized into two functional phenotypes, namely pro‐inflammatory M1 and anti‐inflammatory M2 macrophages [11, 12]. In RA‐affected joints, M1 macrophages are mostly present, which release pro‐inflammatory cytokines and induce the polarization of more macrophages to the M1 phenotype via reactive oxygen species (ROS) and aggravate disease progression [13, 14]. In addition, synovial tissue proliferation and inflammatory cells may lead to hypoxia in the RA synovium [15, 16], which further upregulates inflammatory cytokines and promotes the transformation of macrophages to the M1 phenotype [17, 18]. Therefore, excessive ROS production and a hypoxic environment are the two main factors that hamper the efficacy of RA treatment.

To date, different types of materials, including natural enzymes and artificial mimetic enzymes, have been reported to downregulate ROS levels and alleviate hypoxia in an inflammatory microenvironment [19]. For example, Ping et al. prepared methotrexate and catalase (CAT) co‐encapsulated liposomes, which catalyzed the decomposition of hydrogen peroxide into oxygen (O_2_) and water in the inflammatory environment [20]. However, the stability and high cost of these natural enzymes hamper their applications in this context. To address this, functional nanomaterials with enzyme‐mimetic activities, such as Fe_3_O_4_, MnO_2_, and CeO_2_, have generated considerable interest because of their high stability and controllable catalytic activities, particularly in enhancing efficacy through photothermal effects, ROS clearance, and other mechanisms [21, 22, 23, 24, 25]. Wang et al. prepared Au@CeO_2_ nanorods to inhibit inflammatory cytokine secretion and induce inflammatory cell death via photothermal response and downregulate the expression of hypoxia‐inducible factor through the O_2_‐generating catalytic reaction of ceria (CeO_2_) [25]. The clearance of excessive ROS, such as hydrogen peroxide (H_2_O_2_) and superoxide anions (O_2_•−), can help relieve oxidative stress in inflamed joints and inhibit pro‐inflammatory M1 macrophage‐mediated responses. Recently, Shi et al. reported a manganese porphyrin with both CAT and superoxide dismutase‐like (SOD) activities, which can trigger the transition of macrophages from M1 to M2 phenotype by eliminating ROS in an RA mouse model [26].

The synergistic effect of external physical factors and internal biochemical factors is desirable to achieve more efficient and safe therapeutic outcomes. Nanomaterials based on noble metals, such as gold, silver, ruthenium, platinum, and palladium, are promising candidates for several biological applications because of their intrinsic chemical inertness and unique physical and chemical characteristics [27, 28, 29]. Ruthenium‐based nanomaterials (RuNPs) have been investigated because of their unique biological properties, such as Ru (II)/Ru (III) catalytic activity, ROS downregulation ability, and photothermal effects. These nanomaterials have shown great potential in tumor therapy and have extensive antioxidant/anti‐inflammatory applications [28, 30]. For example, Liu et al. developed novel pompon‐like RuNPs using the polyol reduction method, which had remarkable photothermal properties for simultaneous photothermal therapy (PTT) and photodynamic therapy (PDT) of tumors. Kang et al. prepared speckled Ru–Te hollow nanorods with multiple nanozymatic activities, including peroxidase‐, SOD‐, and CAT‐like activities, which accelerated the accumulative formation of O_2_ and improved the efficacy of PDT for cancer treatment [31]. However, most of the Ru‐based anti‐inflammatory nanoagents have poor catalytic properties and relatively low photothermal conversion efficiency owing to their larger particle size and unsatisfactory dispersity on the support matrix [32]. In addition, the strong photothermal effect of Ru‐based nanoagents could be detrimental because they cannot specifically target M1‐type macrophages. Therefore, it is challenging to construct high‐quality Ru‐based anti‐inflammatory nanoagents with enhanced multi‐enzymatic activities and improved photothermal properties for RA treatment.

**SCHEME 1:**
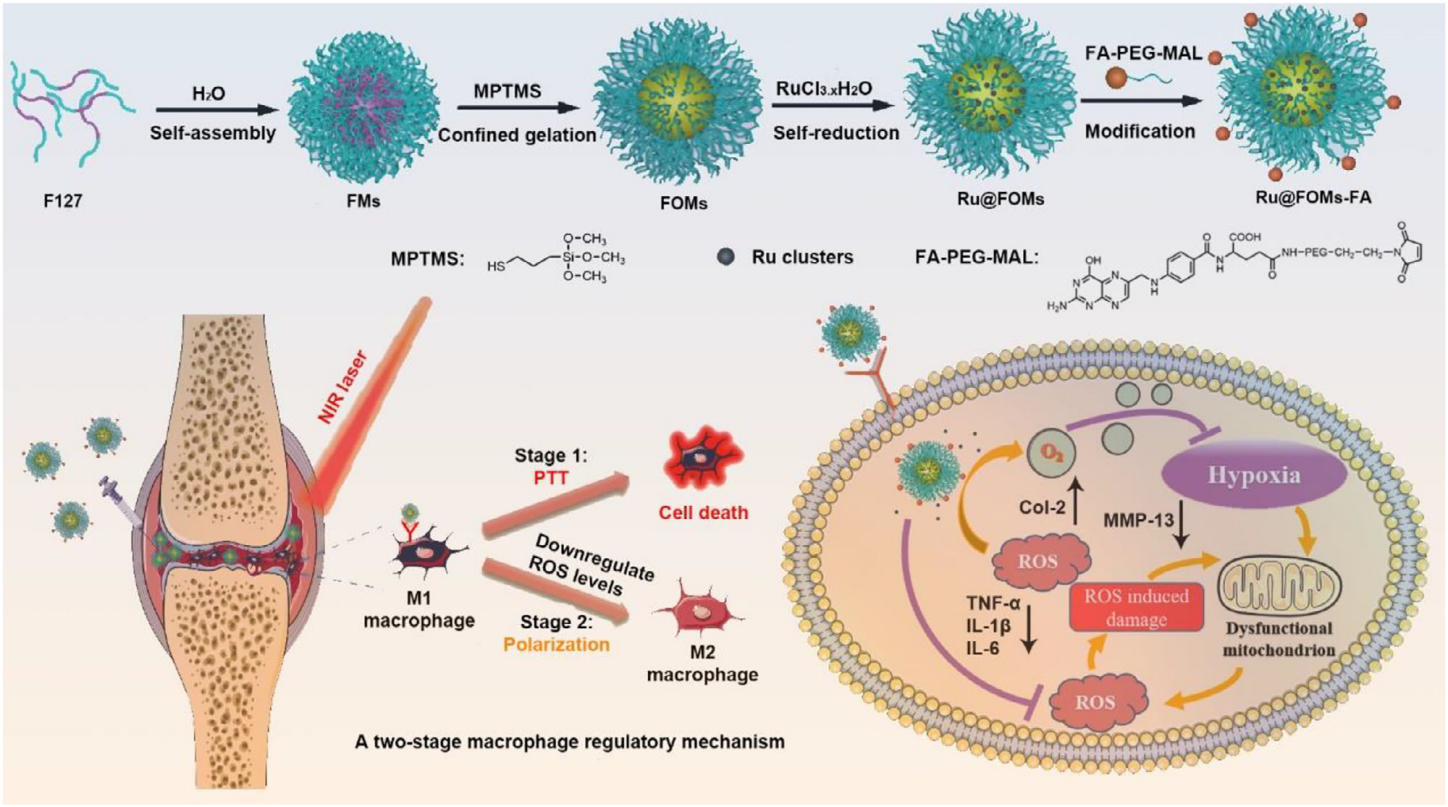
Preparation process of RuFOMs‐FA and their therapeutic mechanism against RA. RuFOMs‐FA actively target inflammatory M1 macrophages through folate receptor (FR‐β) expressed on the macrophage surface. RuFOMs‐FA scavenge ROS and produce O_2_ synergistically, leading to the polarization of pro‐inflammatory M1 to anti‐inflammatory M2 macrophages in hypoxic and inflamed joints. Besides, RuFOMs‐FA induce the death of M1 macrophages through PTT using NIR laser irradiation.

Herein, we reported a novel, targeted ruthenium‐based anti‐inflammatory nanosystem consisting of ruthenium clusters‐loaded F127‐organosilica micelles (FOMs) with folic acid modification (RuFOMs‐FA) for efficient and safe RA treatment. Scheme 1 depicts the preparation of the FOMs, as described in our previous study [33]. Then, ruthenium trichloride salts were self‐reduced to ruthenium nanoclusters in a restricted and disulfide/thiol‐doped organosilica framework of FOMs, resulting in the formation of Ru nanoclusters‐loaded F127 organosilica‐stabilized micelles (RuFOMs). Finally, we introduced FA‐PEG‐MAL molecules to modify RuFOMs through the Michael addition reaction between the MAL group and thiols on the surface of RuFOMs to graft the folic acid. Compared to other support materials, FOMs provided unique confined and stabilized loading spaces for high‐active and ultra‐dispersed metal sites, which can remarkably improve the ruthenium`s photothermal effect and simulated‐enzyme catalytic activity. On the other hand, the inherent functional organosilica surface of FOMs is easier to modify, targeting molecules to bind the specific cells.

Benefiting from the exquisite material design and remarkable photothermal effect and multi‐enzyme catalytic activity of RuFOMs‐FA, a two‐stage macrophage regulatory mechanism was proposed to treat RA (Scheme 1). In the first stage, part of folate receptor (FR‐β)‐expressing M1 macrophages would be killed under external 808 nm laser irradiation in a short time period by RuFOMs‐FA with high photothermal conversion efficiency [34, 35, 36]. In a second stage of internal biochemical regulation, owing to unique CAT‐ and SOD‐like activities of RuFOMs‐FA, the polarization of pro‐inflammatory M1 to anti‐inflammatory M2 macrophages in the RA physiological environment occurred by the steady‐state regulation of ROS levels in the inflamed joints and relieved synovium hypoxia simultaneously by generating massive O_2_. Consequently, this two‐stage regulatory mechanism toward macrophages provides a promising therapeutic strategy in the treatment of RA by a combination of short‐term external physical stimulus and long‐term internal biochemical regulation.

## 2 Results and Discussion

### 2.1 Synthesis and Characterization of RuFOMs‐FA

**FIGURE 1:**
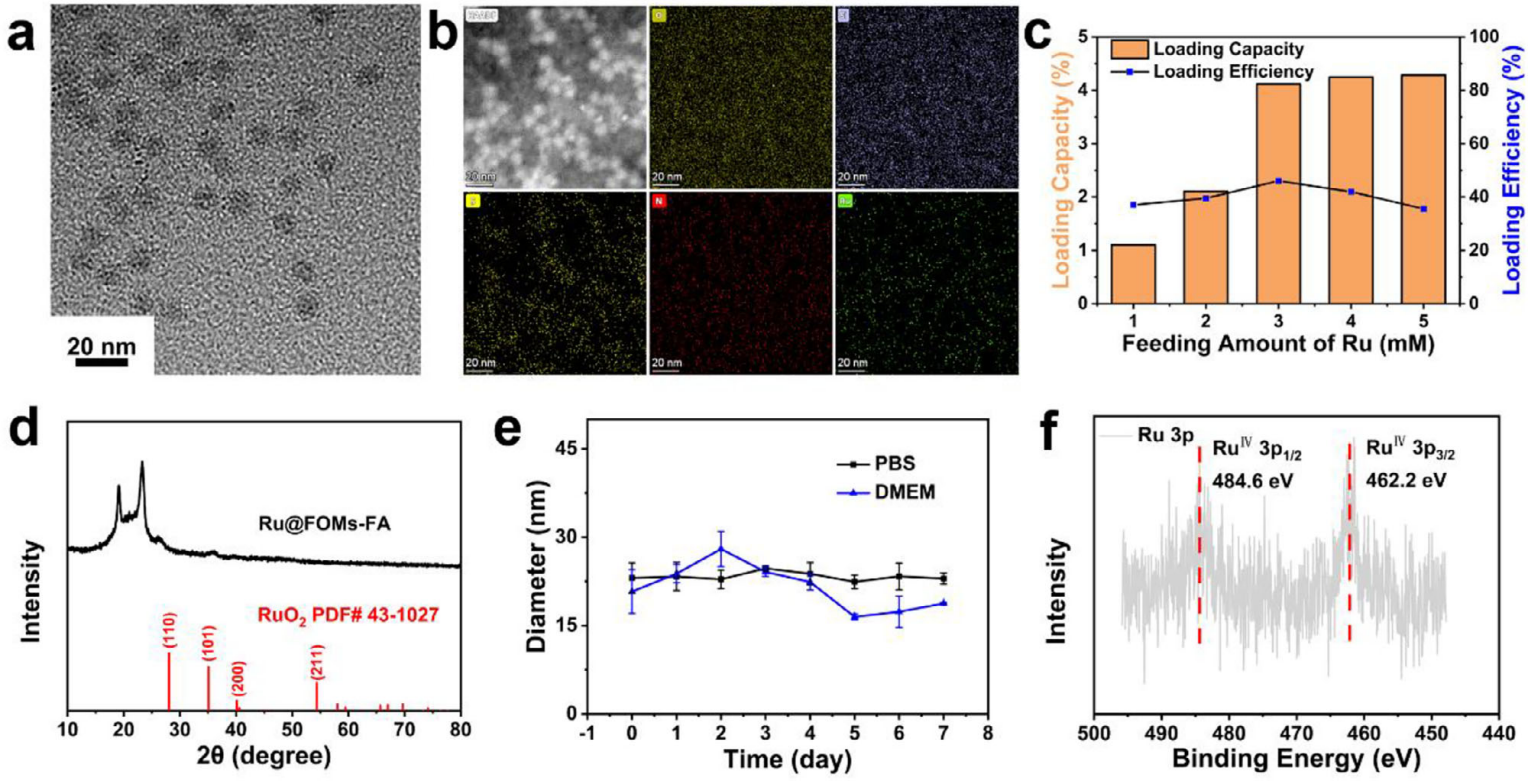
(a) TEM image of RuFOMs‐FA (b) HAADF‐STEM image of RuFOMs‐FA and corresponding element mapping images of O, Si, S, N, and Ru, respectively. (c) Ru loading capacity and loading efficiency of RuFOMs prepared with different amounts of RuCl_3_·*x*H_2_O. (d) XRD pattern of RuFOMs‐FA with the standard JCPDS file (no. 43–1027). (e) Hydrodynamic diameters of RuFOMs‐FA in PBS (pH 7.4) and DMEM in 7 days. (f) XPS spectrum in the Ru 3p region of RuFOMs‐FA.

The RuFOMs‐FA nanoparticles were synthesized through a facile “in situ confined self‐reduction” strategy using RuCl_3_·*x*H_2_O as a ruthenium source and disulfide/thiols‐doped F127‐organosilica micelle as a particle reductive agent. TEM images showed that the particle size of RuFOMs‐FA was approximately 9 nm (Figure 1a), which was smaller than that obtained using the DLS. This is probably due to the low‐contrast PEO blocks extended in the aqueous phase, which were not observed in the TEM images [37]. In contrast, a large number of black dots were seen on the RuFOMs‐FA, which can be assigned to the ultra‐small ruthenium nanoclusters. The corresponding EDS elemental mapping results further confirmed the existence of Ru on RuFOMs‐FA (Figure 1b). To optimize the ruthenium loading, several RuFOMs were prepared by mixing RuCl_3_·*x*H_2_O with various amounts and FOMs. Dynamic light scattering (DLS) showed that the hydrodynamic diameters of RuFOMs increased from 12 to 22 nm as the initial concentration of RuCl_3_·*x*H_2_O changed from 1 to 5 mM (Figure S1a). The size increment is probably due to the increase in the zeta potential of RuFOMs (Figure S1b). The ICP‐MS quantitative results showed that RuFOMs‐3 mM had the highest loading efficiency of 46.1% and a relatively higher loading capacity of 4.1 wt% compared to other RuFOMs samples (Figure 1c). The increased hydrodynamic diameter to 21 nm and a higher negative potential (−6.5 mV) (Figure S2) [38, 39], as well as the decrease of the S─H peak and appearance of the C═O peak in the FT‐IR spectra (Figure S3), verified the successful graft of FA.

X‐ray diffraction (XRD) pattern of RuFOMs‐FA (Figure 1d) indicated that obvious crystallized peaks of Ru were not found, which suggested that the ruthenium species probably existed as amorphous nanoclusters in RuFOMs‐FA. It is noted that the peaks between 15° and 25° is assigned to the crystallization peaks of F127 polymers, as evidenced by the XRD patterns of FMs and FOMs (Figure S4). Besides, the ^29^Si solid NMR spectra of FOMs and RuFOMs have been measured to characterize the chemical structure of the organosilica framework. As shown in Figure S5, the distinct peaks with the chemical shifts of ca. −69 ppm are clearly observed, corresponding to the (Si‐O)_3_Si‐R (T^3^) chemical structure [33]. This indicates the existence of the organosilica framework, which results from the hydrolysis and condensation of MPTMS molecules. The biostability of RuFOMs‐FA was also investigated using DLS. The hydrodynamic diameter of RuFOMs‐FA remained constant at approximately 21 nm after incubating in PBS and DMEM for up to 7 days, suggesting the excellent long‐term stability of RuFOMs‐FA in the biological media (Figure 1e). X‐ray photoelectron spectroscopic (XPS) analysis was used to determine the valence state of Ru in RuFOMs‐FA (Figure 1f and Figure S6). The peaks of C 1s and Ru 3d orbitals overlapped; therefore, the effect of the C 1s peak from F127, which included the C─O peak at 286.0 eV and the C─C peak at 284.6 eV, on Ru was eliminated [40, 41]. After eliminating the impact of the C 1s peak, the binding energies at 285.3 and 281.2 eV were attributed to the 3d_3/2_ and 3d_5/2_ orbitals of Ru^IV^. Moreover, the peaks centered at 484.6 and 462.2 eV referred to the 3p_1/2_ and 3p_3/2_ orbitals of Ru^IV^ of RuFOMs‐FA [42, 43], which further confirmed that the state of ruthenium species in RuFOMs‐FA was RuO_2_ nanoclusters.

### 2.2 Photothermal Performance of RuFOMs‐FA

**FIGURE 2:**
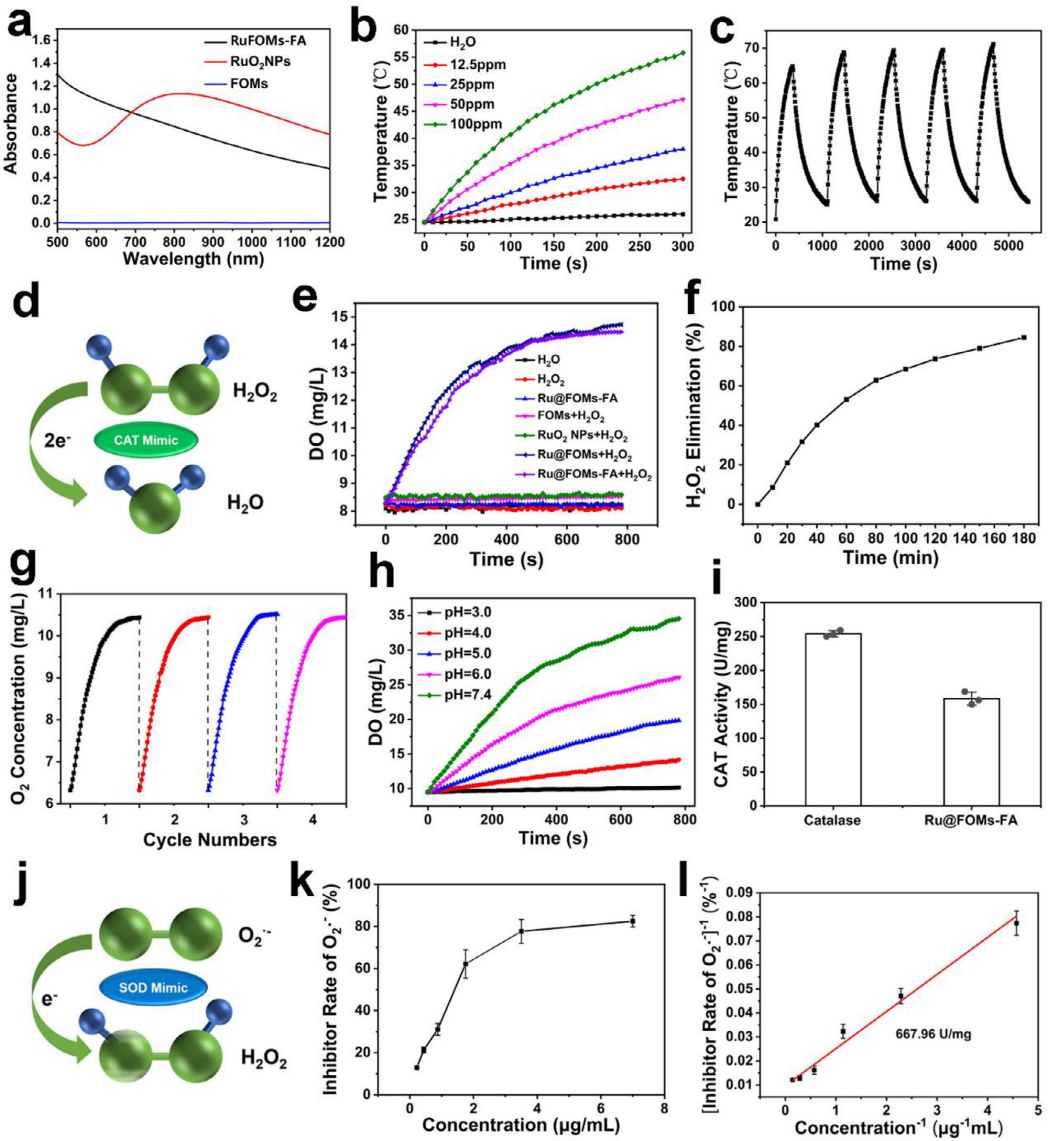
(a) UV–vis spectra of FOMs, RuFOMs‐FA, and RuO_2_ NPs. (b) Temperature elevation curves of RuFOMs‐FA with varying concentrations of Ru upon 808 nm laser irradiation. (c) Photothermal stability of RuFOMs‐FA (100 mg L^−1^ Ru) for five heating–cooling cycles. (d) Schematic diagram of the CAT‐mimetic mechanism of RuFOMs‐FA. (e) Time‐dependent O_2_ production after treating RuFOMs, RuFOMs‐FA, and RuO_2_ NPs with H_2_O_2_ (10 mM) in water. (f) H_2_O_2_‐elimination behavior of RuFOMs‐FA over time. (g) O_2_ concentration in RuFOMs‐FA solution after H_2_O_2_ (10 mM) addition in four cycles. (h) CAT‐mimetic activity of RuFOMs‐FA versus variations in pH. (i) H_2_O_2_ catalytic activity of natural CAT and RuFOMs‐FA. (j) Schematic diagram of SOD‐mimetic mechanism. (k) SOD‐mimetic assay of superoxide anion inhibition with RuFOMs‐FA at varied Ru concentrations. (l) Linear relationship between enzyme activity and inhibition percentage of RuFOMs‐FA.

Ru‐based nanoparticles have been widely used as photothermal agents in tumor therapy [44, 45]. Here, we studied the photothermal properties of RuFOMs‐FA in an aqueous solution. Figure 2a shows that RuFOMs‐FA displays strong absorbance in the NIR region, and its photothermal characteristics are dependent on the ruthenium concentration and laser power density (Figure 2b and Figure S7). Specifically, under 808 nm laser irradiation (1.0 W cm^−2^), the temperature increased from 24.5°C to 56.0°C at the ruthenium concentration of 100 mg L^−1^. Figure 2c shows that the temperature can still increase from 25°C to 70°C even after five irradiation cycles. Moreover, no changes in the UV–vis absorbance spectra of RuFOMs‐FA have been found before and after five irradiation cycles (Figure S8), suggesting the excellent photostability of RuFOMs‐FA. The photothermal conversion efficiency of RuFOMs‐FA was calculated as 55.33% (Figure S9), which was higher than that of other reported metal‐based photothermal agents (Table S1). To further highlight the photothermal property of RuFOMs‐FA, a control sample of pure RuO_2_ NPs was synthesized using the hydrothermal method. XRD pattern (Figure S10) and DLS result (Figure S11) of RuO_2_ showed that the hydrodynamic diameter of RuO_2_ NPs was 8 nm and its crystal structure was rutile RuO_2_ (JCPDS card no. 43–1027). The optical performance experiments showed that although the absorbance intensity of RuO_2_ in the NIR region was stronger than that of RuFOMs‐FA at the same ruthenium concentration of 100 mg L^−1^, its photothermal conversion efficiency (42%) was lower than that of RuFOMs‐FA (Figure S12). Overall, these results indicate that the uniformly distributed ultra‐small RuO_2_ nanoclusters on a stable silica‐based matrix contribute to the enhanced photothermal performance of RuFOMs‐FA.

### 2.3 CAT‐ and SOD‐Like Properties of RuFOMs‐FA

To investigate the CAT‐like property of RuFOMs‐FA (Figure 2d), the time‐dependent O_2_ production and H_2_O_2_ decomposition were measured using a portable dissolved oxygen meter. The O_2_ production increased from 8.30 to 14.46 mg L^−1^ in 13 min in the RuFOMs‐FA plus H_2_O_2_ group, and the graft of folic acid did not significantly interfere with the CAT‐like property of RuFOMs (Figure 2e). In contrast, O_2_ was not produced in the absence of RuFOMs‐FA, RuFOMs, or H_2_O_2_. The control sample of RuO_2_ NPs with larger particle sizes was unable to catalyze the conversion of H_2_O_2_ to O_2_, which, in turn, revealed that the unique CAT‐like property of ultra‐small RuO_2_ nanoclusters on FOMs can be attributed to their good affinity to H_2_O_2_. The UV absorbance at 240 nm decreased over time gradually after incubation with RuFOMs‐FA, and approximately 84.5% of H_2_O_2_ was decomposed in 180 min (Figure 2f and Figure S13), further indicating the CAT‐like property of RuFOMs‐FA. In addition, RuFOMs‐FA showed good cycle stability in catalyzing the conversion of H_2_O_2_ to oxygen (Figure 2g). Moreover, the CAT‐like activity of RuFOMs‐FA increased with an increase in the pH from 3.0 to 7.4 (Figure 2h), indicating the pH‐dependent CAT‐mimic property of RuFOMs‐FA. The CAT‐like activity of RuFOMs‐FA was 62.3% of natural CAT isolated from the beef liver (Figure 2i). The Michaelis constant (*K*_m_) and the maximum reaction velocity (*V*_m_) of RuFOMs‐FA were 0.239 M and 0.584 mg L^−1^ S^−1^, respectively (Figure S14), and the values were comparable to those of other reported noble metal‐based nanozymes and natural catalases (Table S2). Further, the O_2_•^−^ scavenging activity (SOD‐like activity) of RuFOMs‐FA was assessed using the nitro blue tetrazolium (NBT) reduction method (Figure 2j). The O_2_•^−^ inhibition rate was 82.5% at the ruthenium concentration of 7 mg L^−1^, suggesting the efficient SOD‐like enzymatic activity of RuFOMs‐FA (Figure 2k). The catalytic activity of RuFOMs‐FA was 667.96 U mg^−1^ (Figure 2l), which was equivalent to 40.2% of natural SOD (originating from pig blood, Figure S15). These results demonstrated that RuFOMs‐FA exhibited an excellent O_2_•^−^ scavenging capacity and could be used as efficient SOD‐mimic materials.

### 2.4 In Vitro Safety and RA Therapeutic Effects of RuFOMs‐FA

**FIGURE 3:**
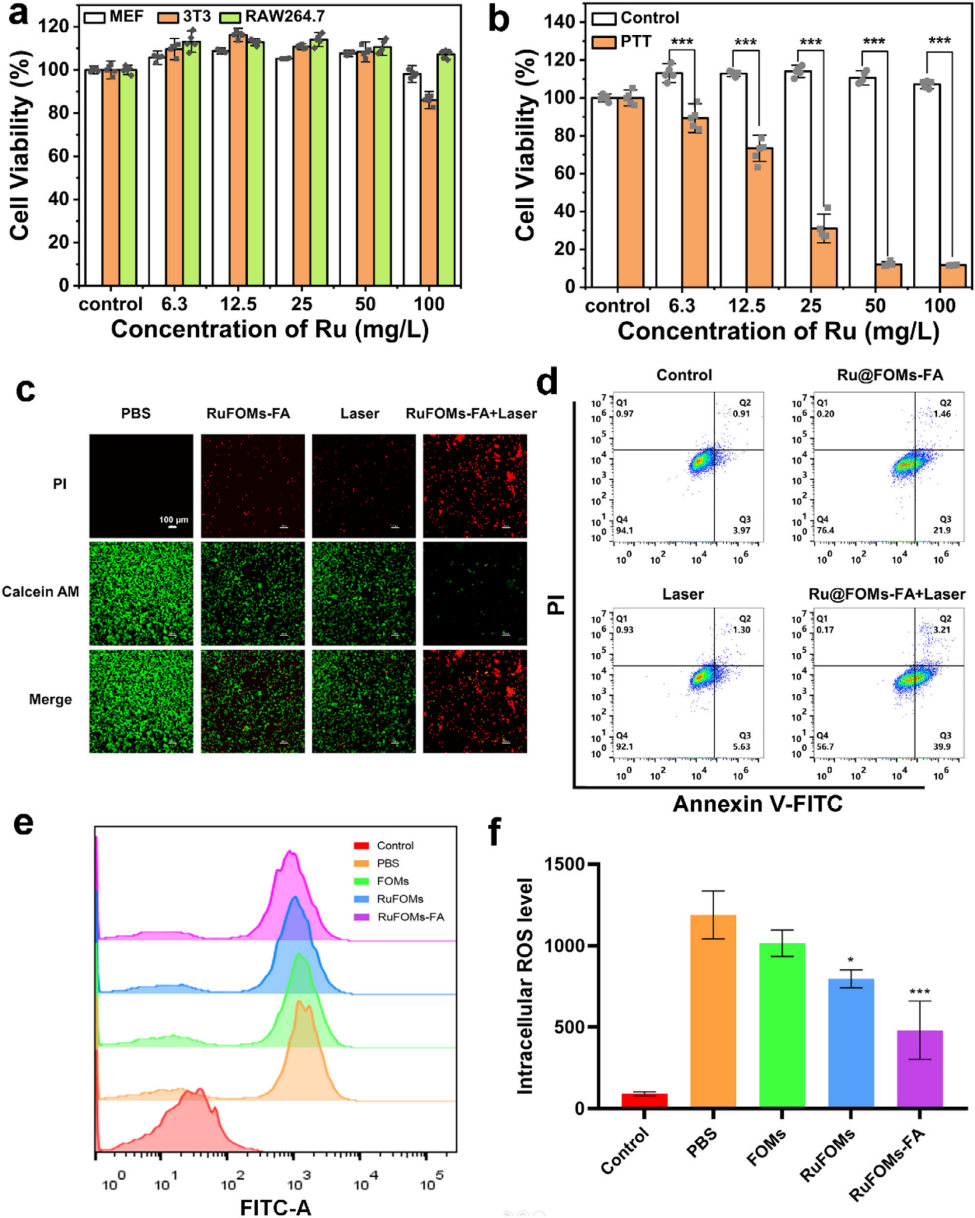
(a) Relative cell viabilities of MEF, NIH‐3T3, and RAW264.7 cells after incubation with RuFOMs‐FA at varied Ru concentrations for 24 h. (b) Relative cell viabilities of RAW264.7 cells incubated with RuFOMs‐FA at varied Ru concentrations under 808 nm laser irradiation (1.0 W cm^−2^). (c) CLSM images of calcein‐AM/PI co‐stained RAW264.7 cells subjected to various treatments (Ru concentration: 100 mg·L^−1^). (d) Apoptosis of RAW264.7 cells after PBS, RuFOMs‐FA, PBS+NIR, and RuFOMs‐FA+NIR incubation was determined using Annexin V‐FITC/PI staining. (e) Flow cytometry analysis and (f) relative fluorescence intensity of intracellular ROS in the RAW264.7 cells treated with different groups. *indicates significant differences compared with the PBS groups (*p* < 0.05). **0.001< *p* < 0.01 versus PBS group; ***p < 0.001 versus PBS group. The data were presented as mean ± SD (*n* = 3).

A mouse monocyte/macrophage cell line (RAW264.7) was used as a cellular model to explore the therapeutic potential of RuFOMs‐FA in RA. First, rhodamine‐labeled RuFOMs‐FA (designated as RhB‐RuFOMs‐FA) were successfully prepared, and their cellular internalization was evaluated using confocal laser scanning microscopy (CLSM) at various time intervals. The red fluorescent signal originating from RhB‐RuFOMs‐FA increased with incubation time, which indicated that RhB‐RuFOMs‐FA entered RAW264.7 cells via the endocytosis pathway (Figure S16). The cytotoxicity of RuFOMs‐FA against mouse embryonic fibroblasts (MEF), mouse embryonic cells (NIH‐3T3 cell line), and RAW264.7 cells using the CCK‐8 assay was evaluated. Cytotoxicity was not detected after incubating these cells with RuFOMs‐FA for 24 h, even at a high ruthenium concentration of 100 mg L^−1^ (Figure 3a). Also, the ROS‐elimination activity and photothermal effect of RuFOMs‐FA at the cellular level were investigated. The in vitro photothermal cytotoxicity of RuFOMs‐FA against RAW264.7 cells was determined under an 808 nm laser irradiation (1.0 W cm^−2^) for 5 min. It was found that RuFOMs‐FA exhibited a concentration‐dependent cell death under laser irradiation (Figure 3b). Notably, the survival rate of RAW264.7 cells decreased to 11.7% ± 0.2% after treatment with RuFOMs‐FA at a Ru concentration of 100 mg·L^−1^.

The fluorescent dyes, calcein‐AM (green) and PI (red), were used to stain the living and dead cells, respectively, to observe cell apoptosis. The red fluorescence corresponding to dead cells was barely visible when there was only RuFOMs‐FA or 808 nm laser irradiation (1.0 W cm^−2^). In contrast, the cells in RuFOMs‐FA plus 808 nm laser group showed strong red fluorescence but weak green fluorescence, thereby demonstrating the remarkable photothermal cytotoxicity of RuFOMs‐FA under laser irradiation (Figure 3c). These results were further confirmed using the flow cytometric analysis. The apoptosis rate of the RuFOMs‐FA plus 808 nm laser group was significantly higher than that of other groups (Figure 3d). The DCFH‐DA reagent was used as a fluorogenic probe to measure the intracellular oxidative stress in the RAW264.7 cells after treatment with specific materials or/and lipopolysaccharide (LPS) to confirm the ROS scavenging capabilities of RuFOMs‐FA. The DCFH‐DA reagent penetrates cell membranes and cleaves into DCFH. Flow cytometry was used to measure the fluorescence intensity (Figure 3e,f). Compared with the PBS group, the overproduced intracellular ROS levels were not suppressed in the FOMs group, whereas a significant ROS scavenging ability was observed in the RuFOMs group, specifically in the RuFOMs‐FA group.

As reported previously [26], LPS induced M1 macrophages to upregulate inflammation by secreting multiple proinflammatory cytokines, such as tumor necrosis factor‐α (TNF‐α), interleukin (IL)‐1 and IL‐6, which caused chondrocyte degeneration. On the other hand, M2 macrophages have anti‐inflammatory activity and tissue remodeling effects. In the present work, to demonstrate the in vitro polarization effect of RuFOMs‐FA on RAW264.7 cells, the immunofluorescence staining was used. As shown in Figure S17, both CLSM images and corresponding quantitative results show that the expression of inducible nitric oxide synthase (iNOS) was significantly downregulated, whereas the expression of Arginase‐1 (Arg‐1) was remarkably upregulated in the RuFOMs‐FA group, compared to the PBS group and other control groups. This indicated that RuFOMs‐FA effectively induced macrophage polarization from M1 to M2 phenotype. Overall, these results demonstrated the remarkable ROS scavenging effects and subsequent macrophage polarization ability of RuFOMs‐FA in vitro.

### 2.5 Therapeutic Efficacy of RuFOMs‐FA in RA In Vivo

**FIGURE 4:**
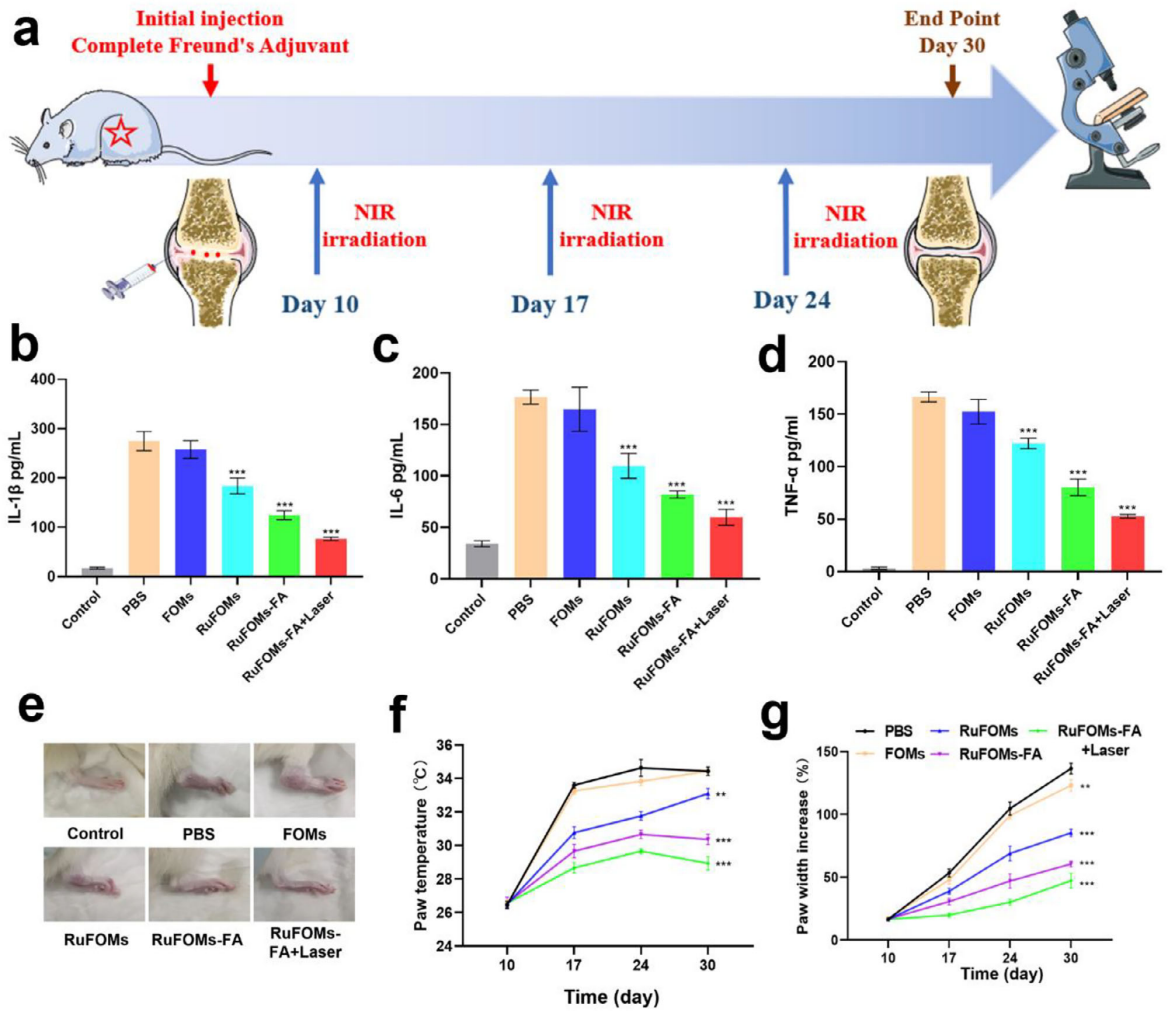
Anti‐inflammatory effects of rat RA knee joints following treatment with various nanoagents. (a) Schematic diagram of in vivo experiments. (b–d) Expression of pro‐inflammatory factors (IL‐1β, IL‐6, and TNF‐α) in the AIA model rats after injecting various materials (as determined using ELISA). (e) Images of the right hind paw and corresponding quantification of (f) paw temperature and (g) paw width at various time points after treatment. *indicates significant differences compared with the PBS groups (*p* < 0.05). **0.001< *p* < 0.01 versus PBS group; ***p < 0.001 versus PBS group. The data were presented as mean ± SD (*n* = 3).

The anti‐inflammatory and cartilage regeneration activity of RuFOMs‐FA was confirmed by assessing the expression of cartilage anabolic and catabolic marker proteins and some key inflammatory cytokines and directly observing histological sections (Figure 4a). Immune cells secrete several inflammatory factors, including tumor necrosis factor (TNF)‐α, interleukin (IL)‐1β, and IL‐6 during the development of RA. Therefore, the expressions of TNF‐α, IL‐1β, and IL‐6 derived from cartilage tissues using an enzyme‐linked immunosorbent assay (ELISA) were evaluated to determine the inflammation‐inhibitory effects of RuFOMs‐FA (Figure 4b–d). The expression of specific pro‐inflammatory cytokines was minimally reduced in the FOMs group compared to that in the PBS group. Further, the expression of pro‐inflammatory cytokines was only partially inhibited in the RuFOMs group. In contrast, the RuFOMs‐FA inhibited the production of pro‐inflammatory cytokines, and the RuFOMs‐FA treated with NIR showed the maximum inhibitory effect on the levels of IL‐1β, TNF‐α, and IL‐6. Notably, the arthritic limb physical function evaluation and thermographic imaging (Figure 4e–g) were consistent with the changes in the expression of inflammatory factors in each group. Overall, the RuFOMs‐FA plus Laser group demonstrated the strongest anti‐inflammatory activity in an RA mouse model.

**FIGURE 5:**
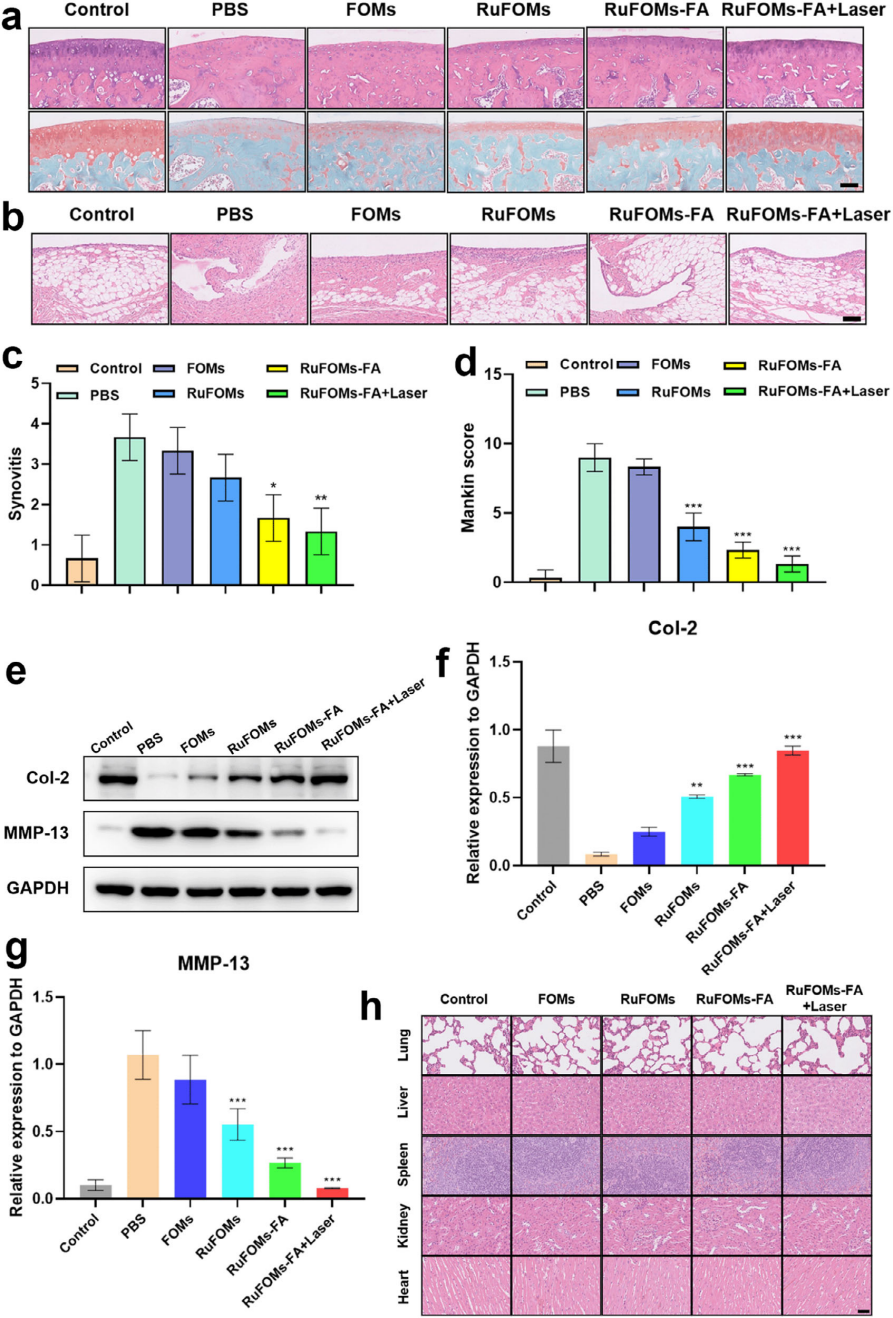
Histological assessments of the rat RA knee joints after treatment. (a) H&E staining (up) and safranin‐O staining (down) of RA‐bearing rats treated with various nanoagents. (b) H&E staining (synovial tissue) and the corresponding quantification of (c) synovitis score and (d) Mankin's score. (e) Western blot analysis and (f,g) corresponding relative quantification of the expression of cartilage anabolic (Col‐2) and catabolic (MMP‐13) markers following the treatment with various groups. (h) H&E staining of the major organs (lung, liver, spleen, kidney, and heart) in the control, FOMs, RuFOMs, RuFOMs‐FA, and RuFOMs‐FA plus Laser groups. *indicates significant differences compared with the PBS groups (*p* < 0.05). **0.001< *p* < 0.01 versus PBS group; ***p < 0.001 versus PBS group. The data were presented as mean ± SD (*n* = 3). Scale bar: 50 µm.

The pathological characteristics of RA, such as cartilage degradation and synovial inflammation in tissue sections, were determined to further assess the therapeutic efficacy of RuFOMs‐FA in the rat RA model [46]. The histological analysis revealed that the progressive degradation of the extracellular matrix (ECM) demonstrated the gradual degeneration of cartilage (Figure 5a) [47]. The tissue sections from the normal group had clean and smooth articular cartilage surfaces with evenly dispersed proteoglycans and collagen type II in the cartilage ECM. However, following the induction of the AIA in the rat model, the articular cartilage surface significantly increased in roughness and irregularity, and a marked infiltration of inflammatory cells was observed. Safranin‐O staining of the articular cartilage in the PBS‐treated group revealed severe cartilage degradation. Moreover, the FOM group demonstrated marked degradation of cartilage. In contrast, these RA‐specific cartilage alterations were partially reversed after intra‐articular injections of RuFOMs, RuFOMs‐FA, and particularly RuFOMs‐FA treated with NIR. The cartilage surface was organized and smooth in the RuFOMs‐FA and RuFOMs‐FA plus Laser groups, which indicated effective preservation of the cartilage structure. In addition, the results of H&E staining and corresponding quantitative analysis (Figure 5b,c) showed that synovitis was significantly reduced in the AIA model rats after the injections of RuFOMs‐FA or RuFOMs‐FA treated with NIR irradiation. Further, RuFOMs‐FA and RuFOMs‐FA plus Laser treatments notably decreased the infiltration of inflammatory cells. Overall, these results suggested that both NIR‐treated and untreated RuFOMs‐FA prevented cartilage destruction.

The cartilage integrity was further evaluated using Mankin's score and the Osteoarthritis Research Society International (OARSI) score (Figure 5d and Figure S18). The results indicated that the best structure was observed in the articular cartilage of the group treated with the RuFOMs‐FA plus Laser, followed by the group treated with RuFOMs‐FA. The effect of RuFOMs‐FA on cartilage protection was then assessed in an RA model using western blotting (Figure 5e–g). Under inflammatory conditions, the expression of the anabolism‐related protein (Col‐2) was significantly decreased, whereas the expression of the catabolism‐related protein (MMP‐13) was markedly increased. However, compared to the PBS group, the expressions of cartilage anabolism marker proteins were significantly upregulated, whereas those of cartilage catabolism marker proteins were significantly downregulated in the RuFOMs‐FA and RuFOMs‐FA plus Laser groups. The results showed that RuFOMs‐FA, particularly RuFOMs‐FA plus Laser group, had a significant protective effect on the cartilage under inflammatory environments. After the treatment, the morphologies of organs, including the lung, liver, kidney, spleen, and heart, were compared between the control, FOMs, RuFOMs, RuFOMs‐FA group, and RuFOMs‐FA plus Laser groups to assess the toxicity of these nanoagents. In addition, no significant histological alterations or morphological changes in all five groups were found, indicating that RuFOMs‐FA treatment was not toxic to the major organs (Figure 5h).

**FIGURE 6:**
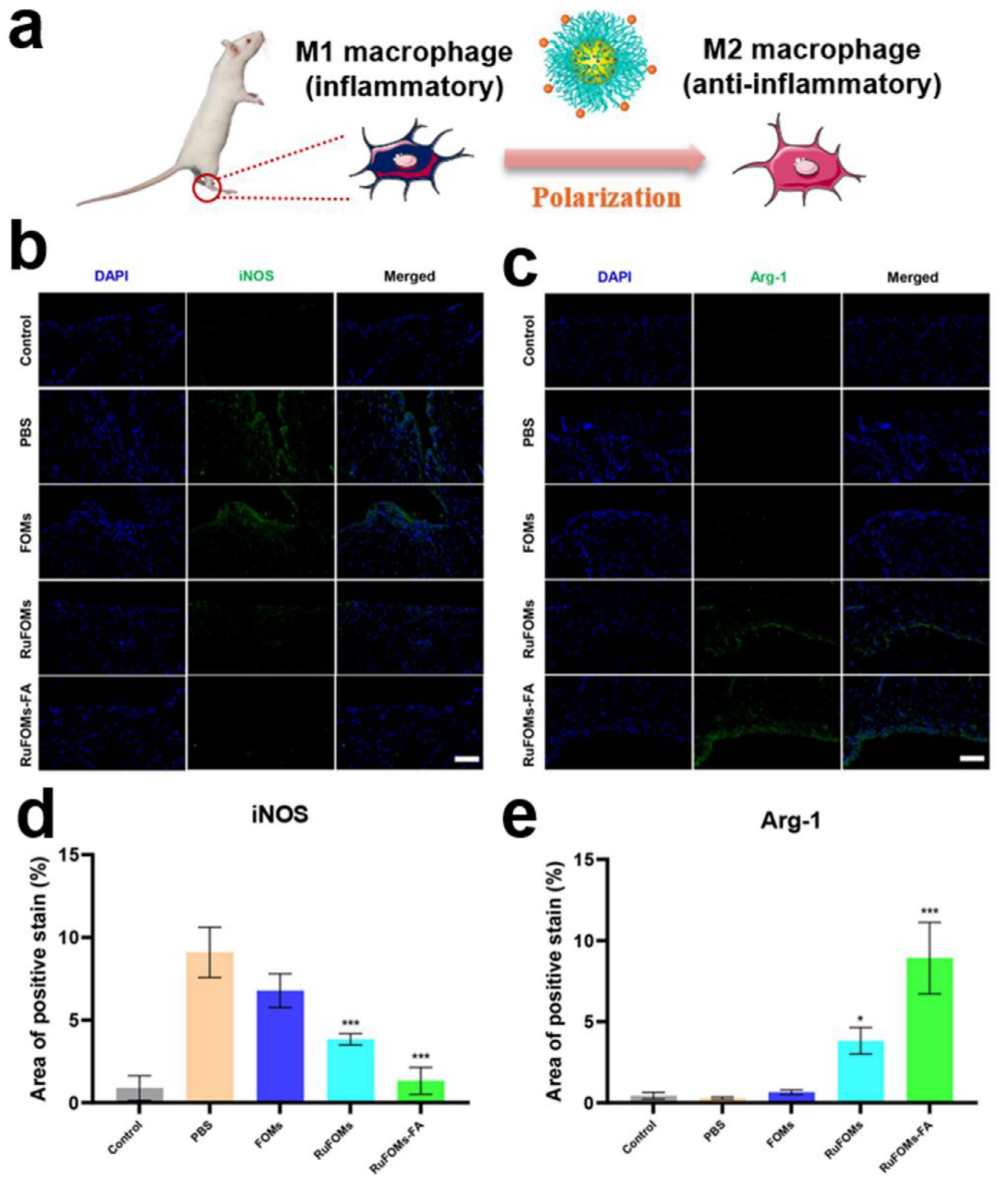
Macrophage polarization ability of RuFOMs‐FA in vivo. (a) Schematic diagram of the mechanism of macrophage polarization. (b,c) Immunofluorescence staining and (d,e) relative fluorescence intensity of the expression of pro‐inflammatory M1‐macrophage biomarker (iNOS) and anti‐inflammatory M2‐macrophage biomarker (Arg‐1) in the synovial tissues of RA rats under various conditions. * indicates significant differences compared with the PBS groups (*p* < 0.05). **0.001< *p* < 0.01 versus PBS group; ***p < 0.001 versus PBS group (*n* = 3).

The phenotypic transition of macrophages is closely related to inflammation in the regional microenvironment [48]. Herein, the immunofluorescence staining assay was used to investigate the polarization effect of RuFOMs‐FA on macrophages (Figure 6a). The CD68 immunofluorescence staining images (Figure S19) show that both bright green and red signals are observed in synovial tissue enriched with macrophages, indicating the good macrophage targeting effect of RuFOMs‐FA in vivo. As shown in Figure 6b–e, the expression of the pro‐inflammatory M1‐macrophage marker (iNOS) was markedly enhanced, whereas the expression of the anti‐inflammatory M2‐macrophage marker (Arg‐1) was significantly reduced in the PBS group. In contrast, the expression levels of iNOS decreased, whereas those of Arg‐1 increased after RuFOMs treatment. Further, the expression of iNOS was downregulated whereas that of Arg‐1 was upregulated in the RuFOMs‐FA group, which indicated the polarization of M1 to M2 macrophages in the RuFOMs‐FA group. Overall, RuFOMs‐FA showed a good anti‐inflammatory effect and induced the phenotypic transition of M1 to M2 macrophages, thereby resulting in their excellent therapeutic efficacy in the rat RA model.

## 3 Conclusions

We designed and developed a targeted ruthenium‐based anti‐inflammatory nanosystem (RuFOMs‐FA) based on the facile preparation of ruthenium clusters‐loaded FOMs and subsequent modification of folic acid for efficient and safe RA treatment. The resultant RuFOMs‐FA had high photothermal conversion efficiency (55.3%) and multiple mimic‐enzyme CAT‐ and SOD‐like activities. These nanoparticles displayed excellent ROS‐scavenging ability and O_2_ production to induce the polarization of M1 to M2 macrophages and exhibited photothermal killing capability toward FA‐overexpressing M1 macrophages in the rat RA models. Cell/animal experiments indicated that RuFOMs‐FA nanoagents are safe and therapeutically effective in rheumatoid arthritis. Therefore, these nanoparticles may be promising metal‐based anti‐inflammatory agents for the treatment of inflammatory diseases.

## Conflicts of Interest

The authors declare no conflicts of interest.

## Supporting information




**Supplementary File 1**: exp270069‐sup‐0001‐SuppMat.docx

## Data Availability

The raw data and processed data required to reproduce these findings are available from the corresponding author upon request.
