# Supplementary material for: Targeted Ruthenium‐Based Anti‐Inflammatory Nanoagent for Enhanced Rheumatoid Arthritis Treatment
Source: Exploration (Beijing). 2025 Aug 16;5(5):20240043. doi: 10.1002/EXP.20240043 (PMC12561266; doi:10.1002/EXP.20240043)
Supplement: Supplementary file 1 — exp270069‐sup‐0001‐SuppMat.docx [file EXP2-5-20240043-s001.docx]

**Supporting information**

**Targeted ruthenium-based anti-inflammatory nanoagent for enhanced rheumatoid arthritis treatment**

Ziwei Zhao^a, 1^, Hao Xiong^b, 1^, Jinyong Wu^a^, Shiyu Xu ^a^, Lihua Zhao ^a^, Yanshuai Wang ^a^, Shuai Chen ^b,^ *, Cunyi Fan ^b,^ *, Dechao Niu ^a,^ *

^a^ Lab of Low-Dimensional Materials Chemistry, Key Laboratory for Ultrafine Materials of Ministry of Education, Frontier Science Center of the Materials Biology and Dynamic Chemistry, School of Materials Science and Engineering, East China University of Science and Technology, Shanghai 200237, China

^b^ Department of Orthopedics, Shanghai Sixth People’s Hospital Affiliated to Shanghai Jiao Tong University School of Medicine, Shanghai, 200233, China.

^1^ These authors contributed equally to this work.

E-mail: [hikev00@163.com](mailto:hikev00@163.com); [cyfan@sjtu.edu.cn](mailto:cyfan@sjtu.edu.cn); [dcniu@ecust.edu.cn](mailto:dcniu@ecust.edu.cn)

**Materials and reagents**

Pluronic®F127 and (3-Mercaptopropyl)-trimethoxysilane (MPTMS) were purchased from Sigma-Aldrich. Folic acid PEG maleimmide (FA-PEG-MAL) was purchased from Beijing Huawei Ruike Chemical Co., Ltd. Ruthenium (III) chloride hydrate (RuCl_3_·xH_2_O) was supplied by Adamas. Ammonia (NH_3_·H_2_O) was purchased from Shanghai Ling Feng Chemical Reagent Co., Ltd. Hydrogen peroxide (H_2_O_2_, 30wt% in water）were bought from Sinopharm hemical Reagent Co., Ltd. Enhanced cell counting kit-8 (CCK-8), catalase and total superoxide dismutase assay kit with NBT were purchased from Beyotime Biotechnology. Catalase (CAT) assay kit was supplied by Nanjing Jiancheng Bioengineering Institute. Superoxide dismutase was purchased from Shanghai Yuanye Biotechnology Co., Ltd. Calcein AM/propidium iodide (PI) Detection Kit, Annexin V-FITC/propidium iodide (PI) Apoptosis Detection Kit, dilactate (DAPI), MAL-PEG-Rhodamine and cell-culture products were all obtained from KeyGEN BioTECH Ltd. All materials and reagents used in experiments did not undergo any purification.

**Characterization and instruments**

The hydrodynamic sizes and zeta potential of samples were measured at room temperature with a Malvern Zeta-sizer Nano Series. Fourier transform infrared (FI-TR) spectra were obtained from Thermo Scientific Nicolet 6700 by using KBr wafer technique. A JEM 2100F electron microscope was used to attain transmission electron microscopy (TEM) images. Element mapping analysis was conducted on a Magellan 400L system (FEI, USA). Ultraviolet-visible absorption (UV-vis) spectra were gotten from spectrophotometer UV-2700 (Shimadzu, Japan). Flow cytometry （FC） was conducted by BD Accuri C6. The Nikon A1 Confocal Laser Scanning Microscopy (CLSM) were used to obtain CLSM images.

**Preparation of FOMs**

Briefly, 600 mg F 127 was dissolved in 10 mL deionized water at 60 ℃, and F127 miccelles (FMs) were obtained. Then 200 μL of NH_3_·H_2_O and 200 μL of MPTMS were slowly added into the FMs solution. Then the solution was stirred at room temperature for 24 h. NH_3_·H_2_O and unreacted MPTMS were removed through dialysis for 24 h. Finally, the F127 organosilica-stabilized micelles (FOMs) were obtained.

**Preparation of RuFOMs**

1.5 mL of FOMs solution and 2 mL of RuCl_3_·xH_2_O solution with a series of defined concentration (5 mM, 10 mM, 15 mM, 20 mM, 25 mM) were mixed in 6.5 mL deionized water under stirring at room temperature for 24 h. Then the sample was dialyzed for 24 h against water to remove free Ru^3+^ and after that the RuFOMs were obtained.

**Preparation of RuFOMs-FA**

Equal amount of RuFOMs and FA-PEG-MAL were blended in deionized water under stirring at room temperature for 12 h. then unreacted FA-PEG-MAL was removed through dialysis against water for 24 h to obtain RuFOMs-FA.

**Preparation of RuO_2_ nanoparticles**

Hydrothermal method was used to obtain RuO_2_ nanoparticles. 210 mg RuCl_3_·xH_2_O was dissolved in 50 mL deionized water which was then stirred for 30 min. The solution was heated to 180 ℃ for 24 h. The products were then collected by centrifugation and washed with ethanol twice followed by drying in a vacuum oven at 35 ℃ for 24 h. At last, products were dispersed in water.

**Stability test of RuFOMs-FA**

The RuFOMs-FA were dispersed into PBS and DMEM medium respectively. The mixture was stored in a shaker under 37 ℃ for 7 days, and the hydrodynamic sizes of the RuFOMs-FA were measured every 24 h.

**Loading efficiency and content of RuFOMs-FA**

The Ru concentration in RuFOMs-FA was measured by Inductively Coupled Plasma Atomic Emission Spectrometer (ICP-MS, 167nm-785nm/725). Loading efficiency (*RuLE*) and loading content (*RuLC*) of Ru were calculated based on eq 1 and 2

$$RuLE=\frac{weight of Ru loaded on micelles}{weight of Ru in feed}\times100\%$$

(1)

$$RuLC=\frac{weight of Ru loaded on micelles}{weight of RuFOMs}\times100\%$$

(2)

**Photothermal effect of RuFOMs-FA**

The photothermal behavior of RuFOMs-FA solution was characterized by using 808 nm NIR laser. And the temperature was recorded digitally throughout the process. Briefly, RuFOMs-FA solution with a series of defined concentration of Ru (100, 50, 25, 12.5, 0 mg/L) was irradiated by 808 nm laser which is fixed to 1 W/cm^2^. Similarly, 100 mg/L of Ru in RuFOMs-FA was tested by different power density (1.5 W/cm^2^, 1 W/cm^2^, 0.75 W/cm^2^, 0.5 W/cm^2^, 0.25 W/cm^2^).

For the photothermal stability of RuFOMs-FA, 100 mg/L of Ru in RuFOMs-FA solution was irradiated by 808 nm NIR laser (1 W/cm^2^) for 5 min. After cooling to room temperature naturally without irradiation, switch on the laser again to irradiate the sample for another 5 min. Repeat the process for 5 times.

To obtain the photothermal conversion efficiency (*η*), 1 W/cm^2^ of 808 nm laser was used to irradiate 100 mg/L Ru in RuFOMs-FA to reach the maximum temperature (*T_max_*) and followed by turning off the laser to let the sample cooling to room temperature to obtain cooling curve. The *η* was obtain from eq 3

$$\eta=\frac{hS\left( T_{max}-T_{surr} \right)-Q_{Dis}}{I(1-{10}^{-A_{808}})}$$

（3）

where $h$ is heat-transfer coefficient, $S$ is surface area of the container, $T_{max}$is the maximum temperature, $T_{surr} \mathrm{is}$ambient temperature, $Q_{Dis}$ is the energy emitted from the quartz dish （0.0168）when irradiated by 808 nm laser, $I$is the laser power density, $A_{808}$ is UV-vis absorption value of RuFOMs-FA which is fixed to 100 mg/L of Ru at 808 nm.

*hS* is calculated based on eq 4

$$hS=\frac{M_{D}C_{D}}{\tau_{s}}$$

（4）

And $\tau_{s}$ is calculated according to eq 5 and 6

$$\theta=\frac{T-T_{surr}}{T_{max}-T_{surr}}$$

（5）

$$t=-\tau_{s}ln\theta$$

（6）

where $\tau_{s}$ is the sample time constant from the cooling curve, $M_{D}$ is the mass of used RuFOMs-FA in the procedure which is 1.0 g, $C_{D}$ represents heat capacity of water that is 4.2 J/g.

**Detection of oxygen production by RuFOMs-FA**

Dissolved oxygen analyzer was used to detect oxygen generation by RuFOMs-FA. RuFOMs-FA solution was poured into 15 mL deionized water containing 150 mmol H_2_O_2_ to make Ru concentration 1 mg/L. Oxygen concentration was recorded every 10 seconds. To make contrasts, Solutions that are deionized water, defined concentration of H_2_O_2_，RuFOMs-FA solution，RuFOMs-FA+H_2_O_2_, FOMs+H_2_O_2_, RuO_2_NPs+H_2_O_2_, RuFOMs+H_2_O_2_ were also monitored. All H_2_O_2_ used were the same concentration.

To investigate the effect of pH on oxygen producing ability of RuFOMs-FA，a series of different solution (pH=3.0, 4.0, 5.0, 6.0, 7.0）were used. And the concentration of RuFOMs-FA was the same.

**Depletion of H_2_O_2_ by RuFOMs-FA**

Solution containing 20 mM H_2_O_2_ was poured into RuFOMs-FA to make Ru 0.1 mg/L. the UV-vis absorption of solution mentioned was tested from 230 nm to 250 nm every 10 minutes until H_2_O_2_ was depleted completely.

**Michaelis-Menton equation of mimicking CAT**

Fixed concentration of Ru in RuFOMs-FA (1 mg/L) and different concentration of H_2_O_2_ (5, 10, 20, 30, 40, 50 mM) were mixed to monitor oxygen production. Michaelis constant *K_m_* and maximum velocity *V_max_* were calculated according to eq 7 and 8

$$V_{0}=\frac{V_{max}[S]}{K_{m}+[S]}$$

(7)

$$V_{0}^{-1}=\frac{K_{m}}{V_{\max}[S]}+\frac{1}{V_{max}}$$

(8)

where *V_0_* is initial reaction rate, [S] represents the concentration of H_2_O_2_.

**Celluar uptake of the RuFOMs-FA**

MAL-PEG-Rhodamine which can be excited by 561 nm laser and emitted at 595 nm was used to graft to RuFOMs-FA to generate fluorescent signals which can be detected by CLSM. RAW 264.7 cells were cultured in DMEM supplemented with 10 % fetal bovine serum at 37 ℃ under 5 % CO_2_. Then 104 cells per dish were cultured in CLSM dish for 12 h to let cells adhere. After that DMEM was replaced by fresh DMEM containing Rhodamine B labelled RuFOMs-FA. After 4 hours, cells were washed 3 times with PBS followed by stained with DAPI for 10 min.

**Cell toxicity in Vitro**

RAW 264.7 cells, NIH 3T3 cells or MEF cells (5000 cells per well, 100 μL) were seeded in 96-well plates for 24 h. And then the cell was incubated with different concentration of RuFOMs-FA (100, 50, 25, 12.5, 6.3, 0 mg/L of Ru) for 24 h. after being washed twice with PBS, the cell viability was evaluated by CCK-8 assay.

To evaluate photothermal effect of RuFOMs-FA on RAW 264.7 cells, DMEM containing a series of defined concentration (Ru=100, 50, 25, 12,5 6.3, 0 mg/L) were incubated with RAW 264.7 cells for 4 h. And 808 nm laser (1 W/cm^2^) was employed to irradiate each well for 5 minutes. After that, cells were incubated for another 20 h followed by washing twice with PBS. The cell viability was evaluated by CCK-8 assay.

**Living and death cell staining**

Calcein-AM and PI assay was employed to stain living cells and dead cells. 10^5^ of RAW 264.7 cells were cultured in CLSM dish for 24 h. after adhesion, DMEM containing RuFOMs-FA (100 mg/L Ru) was used to culture cells. 4 hours later, 808 nm laser (1 W/cm^2^) was used to irradiate cells for 5 min, and then calcein-AM and PI were added to evaluate the photothermal effect. Cells treated with DMEM only or without irradiation or without both were used as control.

**Flow cytometry**

RAW 264.7 cells were culture in 6-well plate with DMEM for 24 h until cells adhered to the plate. Then RuFOMs-FA (100 mg/L of Ru) was poured into the plate. After 4 h, 808 nm laser (1 W/cm^2^) was employed to irradiate for 5 min and cells were then cultured at 37 ℃ for 20 h. cells without irradiation, cells without RuFOMs-FA or cells without both were also undergone the procedure mentioned above as the contrast. Similarly, all cells were collected and washed and stained with Annexin V-fluorescein isothiocyanate isomer (FITC) and PI which then were analyzed by flow cytometry at last.

RAW264.7 was incubated with FOMs, RuFOMs, and RuFOMs-FA for 1 h, respectively, and then cells were treated with 1 μg/ml LPS for 6 h. Next, DCFH-DA reagent (Sigma) was used to incubate with RAW264.7 for 30 min. Finally, RAW264.7 was analyzed via flow cytometry (Beckman Coulter, Fullerton, CA, USA).

**Immunostaining in vitro**

RAW264.7 was incubated with FOMs, RuFOMs, and RuFOMs-FA for 1 h, respectively, and then cells were treated with 1 μg/ml LPS for 6 h. Then, RAW264.7 were stained with anti-iNOS (Abcam, ab15323) and anti-Arg-1 (Abcam, ab91279). Furthermore, the samples were stained with secondary antibodies and cell nuclei were stained using DAPI (Thermo Fisher Scientific).

**Adjuvant-induced arthritis (AIA) model induction and intra-articular injection in vivo**

Rats (Sprague Dawley, 10 weeks old) were procured from Shanghai Sixth People's Hospital and treated in accordance with the customary protocols endorsed by the ethics committee of Shanghai Sixth People's Hospital (DWLL2023-0563). Continuous inhalation of isoflurane was used to induce general anesthesia for the duration of all animal studies. First, as previously described, an AIA model was produced by injecting complete Freund's adjuvant (Chondrex, WA, 10mg/mL) tail-based intradermally. Five groups were randomly selected from the AIA model rats. At day 8, the first time that inflammation was noticed, the RA rats were assigned at random. A single intra-articular injection of 50 µL of PBS, FOMs (200 ppm), RuFOMs (200 ppm), and RuFOMs-FA (200 ppm) was delivered to the RA knee joint at day 10 after RA was induced. After the injection, the RuFOMs-FA+Laser group was treated with NIR irradiation at a power of 1 W/cm^2^ and 5 min once a week. At day 30, overdose anesthesia was used to euthanize all the groups.

Evaluation of the physical function of an arthritic limb: Following AIA model induction, each RA rat had its paw width measured using a caliper until day 30 after complete Freund's adjuvant administration. In the meantime, an infrared thermal imaging device (FLIR i2, FLIR Systems Inc., OR) was used to measure the temperature of the hind paws of RA rats. In order to gauge paw width and temperature, both hind paws of RA rats were measured.

**ELISA analysis**

A mortar and liquid nitrogen were used to grind the femur parts from the RA knees. The samples were then homogenized in 1 mL of PBS using an electric homogenizer before being centrifuged at 10,000 g for 15 min at 4 °C. Following the manufacturer's instructions, ELISA kits were used to quantify the expression levels of IL-1β (Enzolife, ADI-900-131A), TNF-α (Enzolife, ADI-900-086A), and IL-6 (Antigenix, RRF600CKC) in the collected homogenates. A microplate reader (Bio-Rad, Hercules, CA, USA) was used to measure the absorbance at 450 nm.

**Immunofluorescence analysis in vivo**

The characteristics of RA, such as inflammation and macrophage phenotypic transition, were confirmed using immunohistochemical analysis. The samples were placed in the orbital shaker with an EDTA decalcifying solution after being fixed in 4% paraformaldehyde for 7 days at 4 °C. The specimens were fixed in paraffin and cut into 7 μm sections using sagittal section after being decalcified for 3 weeks at room temperature. The sections were dewaxed and stained with anti-CD68 (Abcam, ab31630), anti-iNOS (Abcam, ab15323) and anti-Arg-1 (Abcam, ab91279). Furthermore, the samples were stained with secondary antibodies before being stained with DAPI and scanned using a digital slide scanner (panoramic MIDI, 3DHISTECH).

**H&E staining and Safranin-O staining**

Hematoxylin-eosin (H&E) staining and Safranin-O/fast green staining were used to assess the features of RA, including synovial hyperplasia and cartilage degradation. The sections were stained with hematoxylin solution for 10 min for the H&E staining, which was followed by washing under running water and 95% alcohol. Afterwards, the samples were stained for 3 min with 1% Eosin Y solution. In order to specifically detect proteoglycan in cartilage, the Safranin-O staining was carried out in accordance with the previously described protocol [1, 2]. The sections were first stained for 10 min with Weigert's hematoxylin working solution, followed by a counter-stain for 5 min with the fast green solution and a 1% acetic acid rinse. The 0.1% Safranin O solution was used to further stain the tissue sections for 5 min. To assess the cartilage deterioration and synovial hyperplasia in the joint cavity, respectively, the slides were evaluated using an Olympus DP70 inverted microscope (Japan). Additionally, as previously described, Mankin's score and OARSI score was utilized to assess cartilage degradation [3].

**Western blot analysis**

Following the sacrifice of the rats, RIPA (Beyotime, China) was used to extract proteins according to the manufacturer's instructions. Furthermore, the BCA Protein Assay Kit (Beyotime, China) was used to quantify protein concentration, and Western blot analysis was performed as previously described [4]. Western blot analysis was performed using anti-MMP-13 (Abcam, ab39012) anti-Col-2 (Abcam, ab34712), and anti-GAPDH (Abcam, ab8245).

**Statistical analysis**

All results are presented as the mean ± standard deviation. SPSS statistics (version 22.0, IBM) was used for the statistical analysis, which was based on one-way analysis of variance (ANOVA) with a Tukey post hoc method. When the *p*-value was less than 0.05, the difference was considered statistically significant.


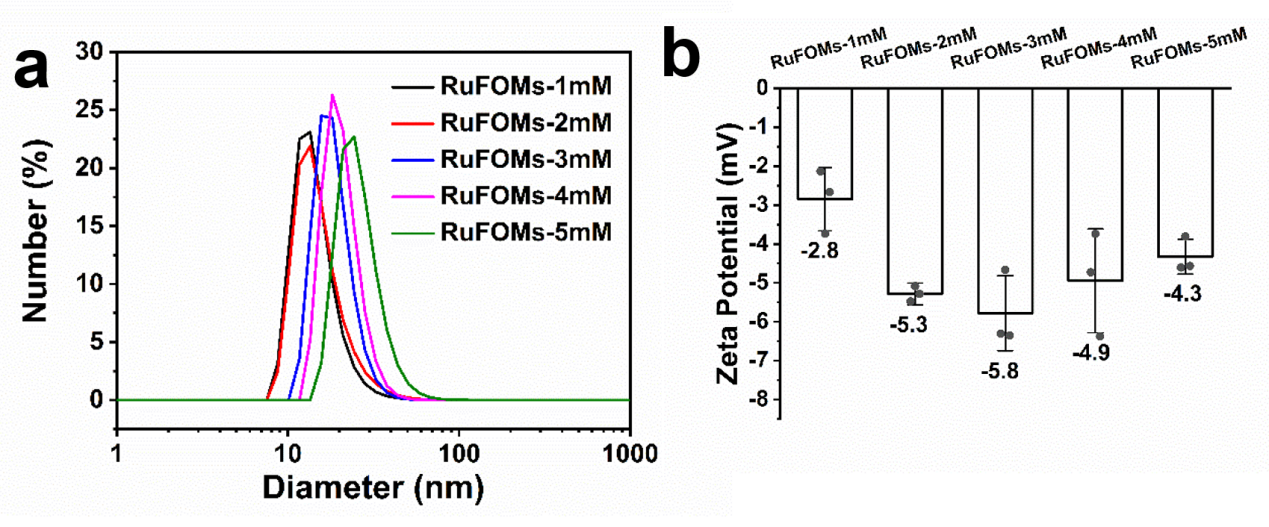


**Figure S1.** (a) Hydrodynamic diameters, (b) Zeta potentials of RuFOMs prepared with different amounts of RuCl_3_·xH_2_O. The used solvent is pure water.


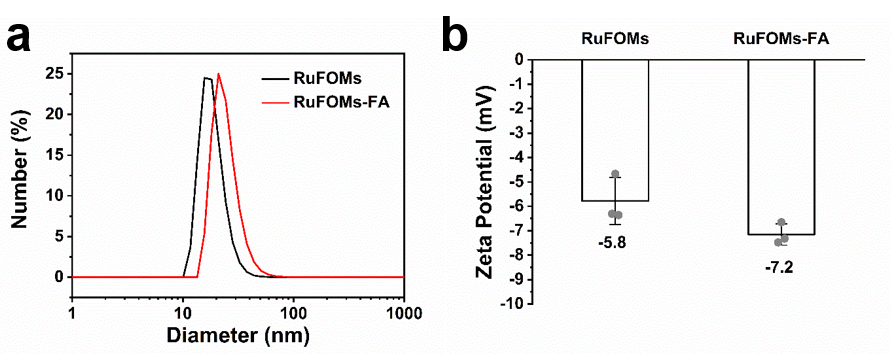


**Figure S2.** (a) Hydrodynamic diameters and (b) Zeta potentials of RuFOMs prepared with 3 mM RuCl_3_·xH_2_O before and after grafted with folic acid. The used solvent is pure water.





**Figure S3.** FT-IR spectra of FOMs, RuFOMs and RuFOMs-FA. The peaks at 2555 cm^-1^ and 1702 cm^-1^ can be attributed to the stretching vibrations of S-H and C=O.


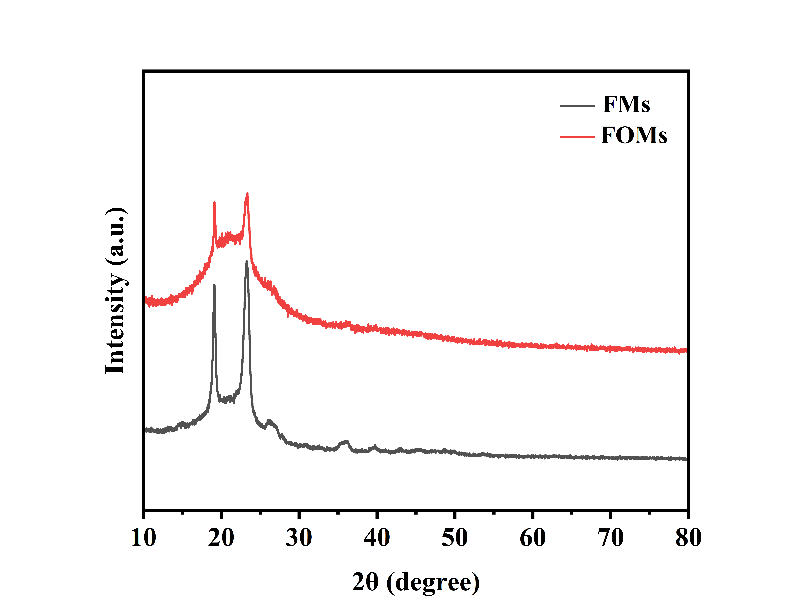


**Figure S4.** XRD patterns of FMs and FOMs.


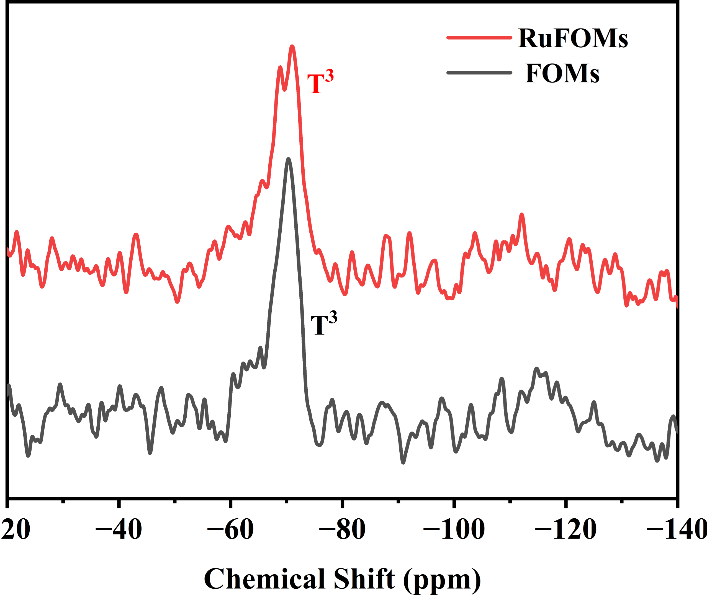


**Figure S5.** The ^29^Si MAS NMR spectra of FOMs and RuFOMs.





**Figure S6.** XPS spectrum in the Ru 3d region of RuFOMs-FA.


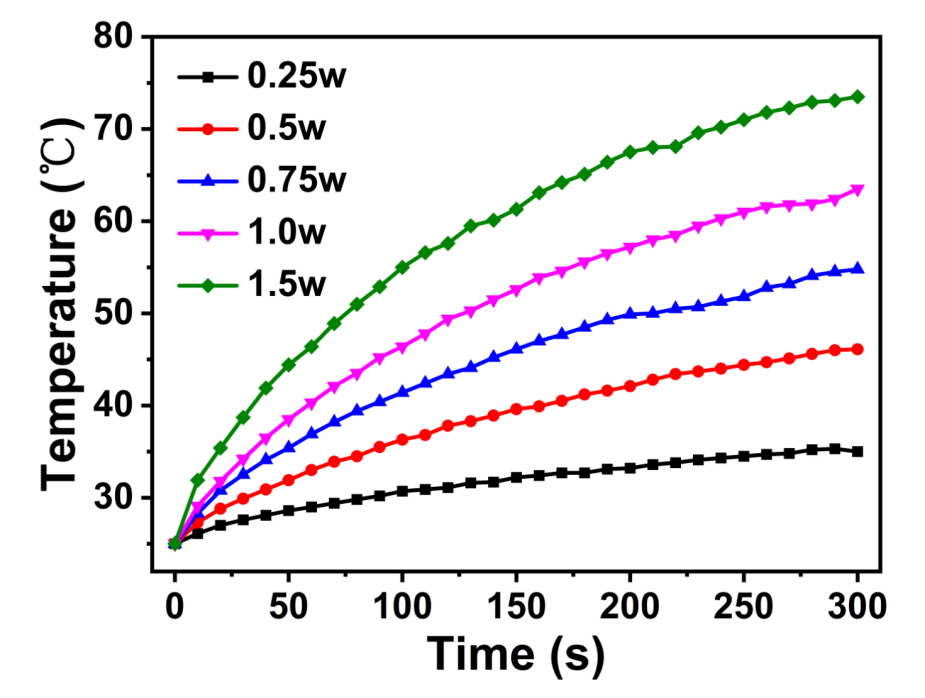


**Figure S7.** Temperature elevation curves of RuFOMs-FA with varying laser power density upon 808 nm laser irradiation.


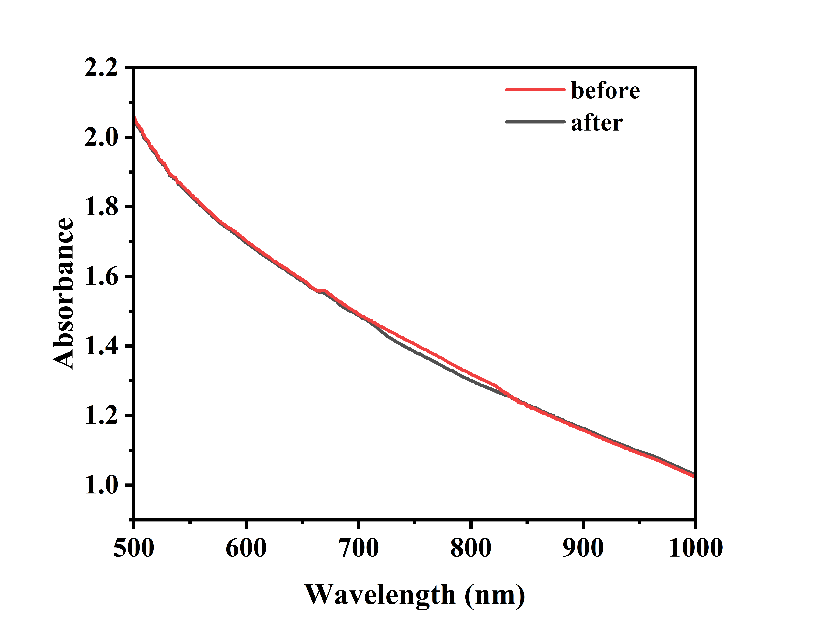


**Figure R8.** The UV-vis absorbance spectra of Ru@FOMs before and after five irradiation cycles.


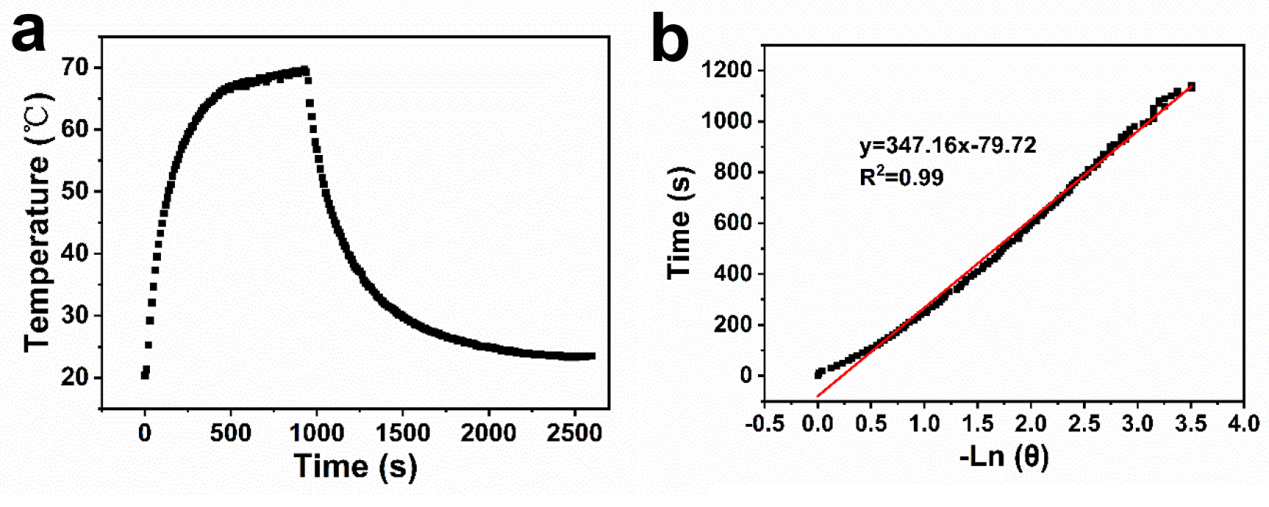


**Figure S9.** (a) Photothermal responses of RuFOMs-FA (100 mg/L Ru) upon irradiation for 15 min before the 808 nm laser (1.0 W cm^-2^) was turned off. (b) Linear time data obtained from a cooling stage (after 15 min).





**Figure S10.** XRD pattern of RuO_2_ NPs with the standard JCPDS file (No.43-1027).


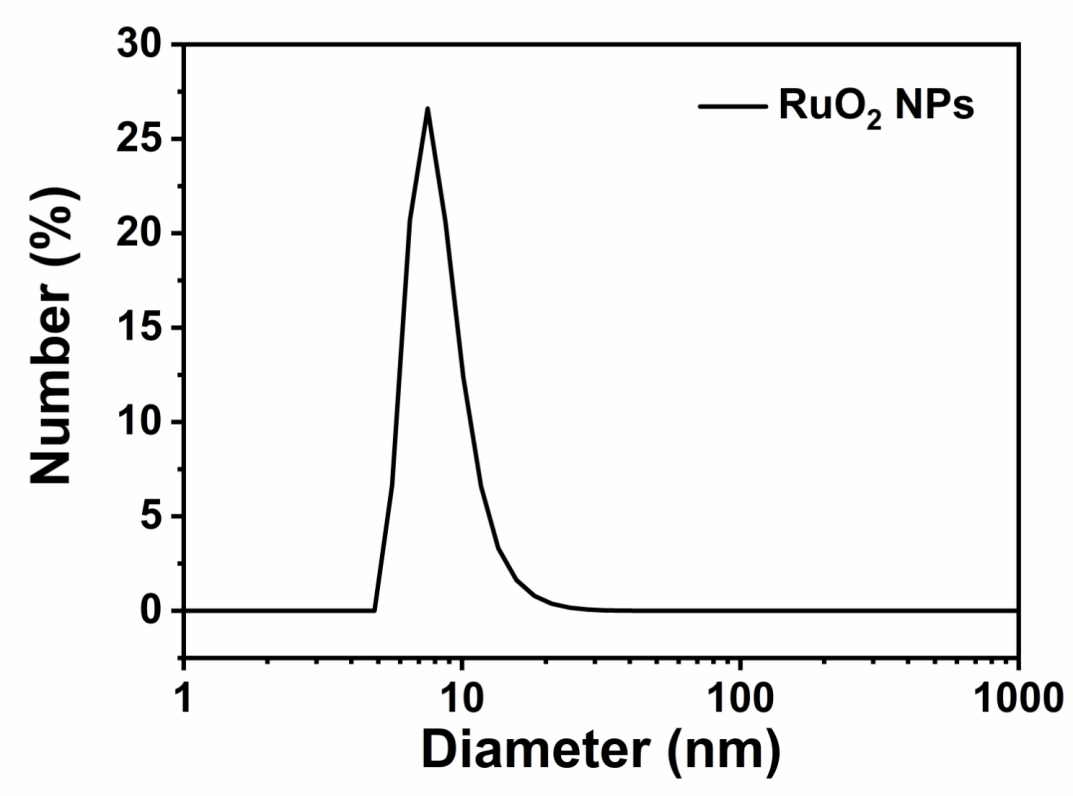


**Figure S11.** Hydrodynamic diameter of RuO_2_ NPs in pure water.


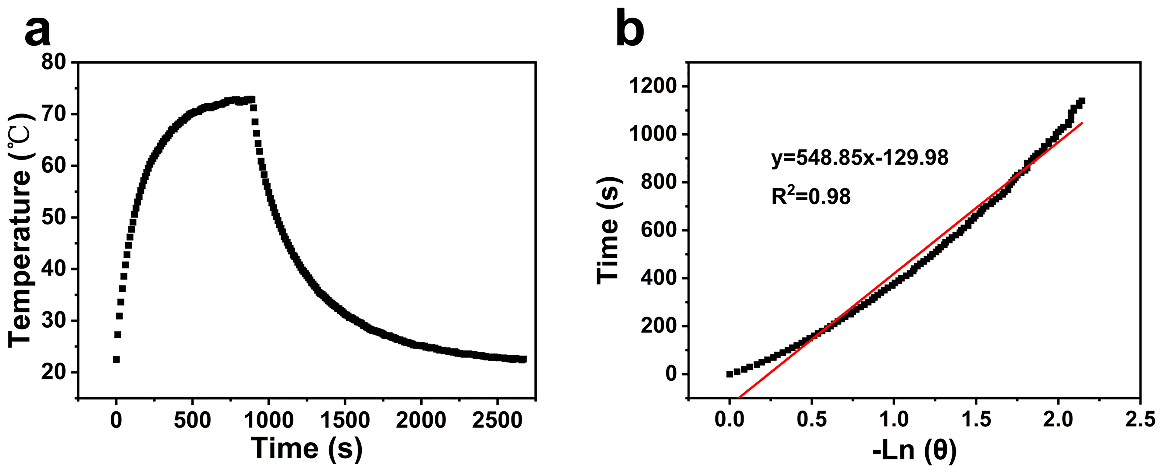


**Figure S12.** (a) Photothermal responses of RuO_2_ NPs (100 mg/L Ru) upon irradiation for 15 min before the 808 nm laser (1.0 W cm^-2^) was turned-off. (b) Linear time data obtained from a cooling stage (after 15 min).





**Figure S13.** H_2_O_2_ (20 mM) consumption over time in the presence of RuFOMs-FA (0.1 mg/L Ru).


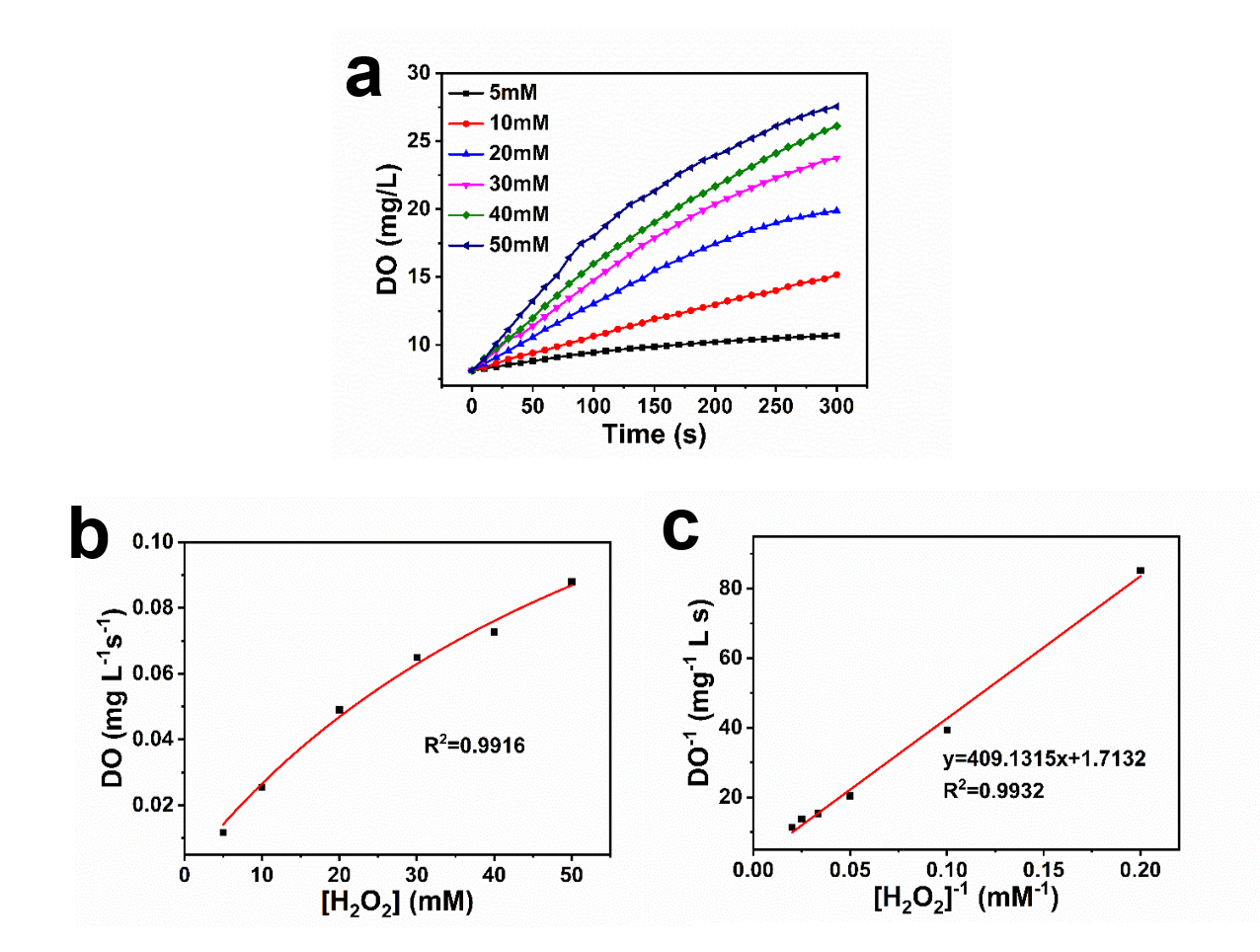


**Figure S14.** (a) CAT-mimetic assay to detect O_2_ production at varied H_2_O_2_ concentrations with RuFOMs-FA. (b) Corresponding Michaelis–Menten equation and (c) Lineweaver–Burk fitting.


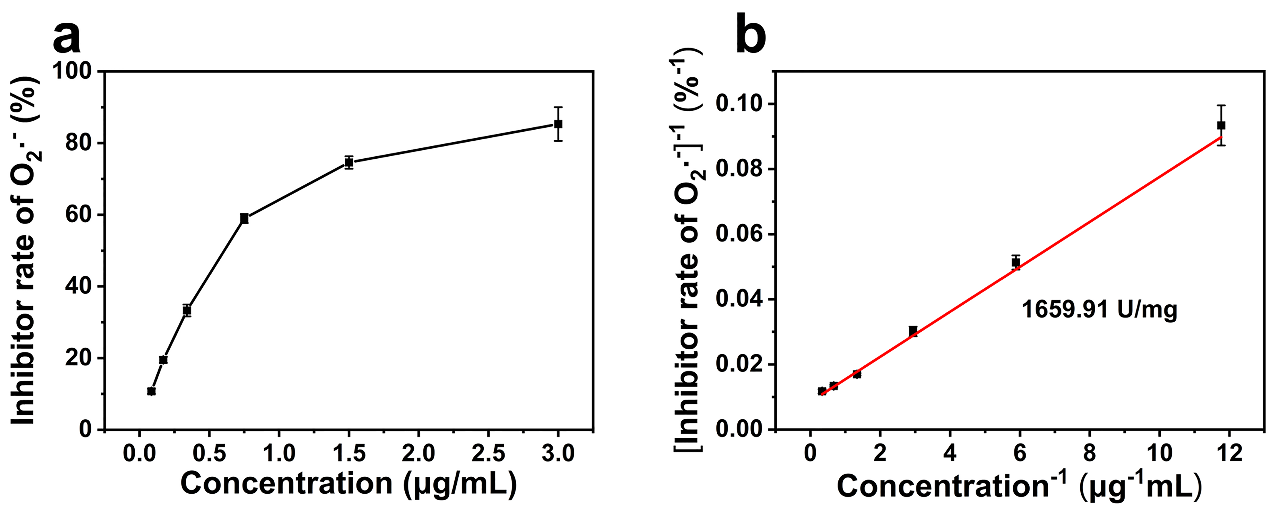


**Figure S15.** (a) Superoxide anion inhibition at varied natural SOD concentrations. (b) The linear relationship between enzyme activity and inhibition percentage of natural SOD.


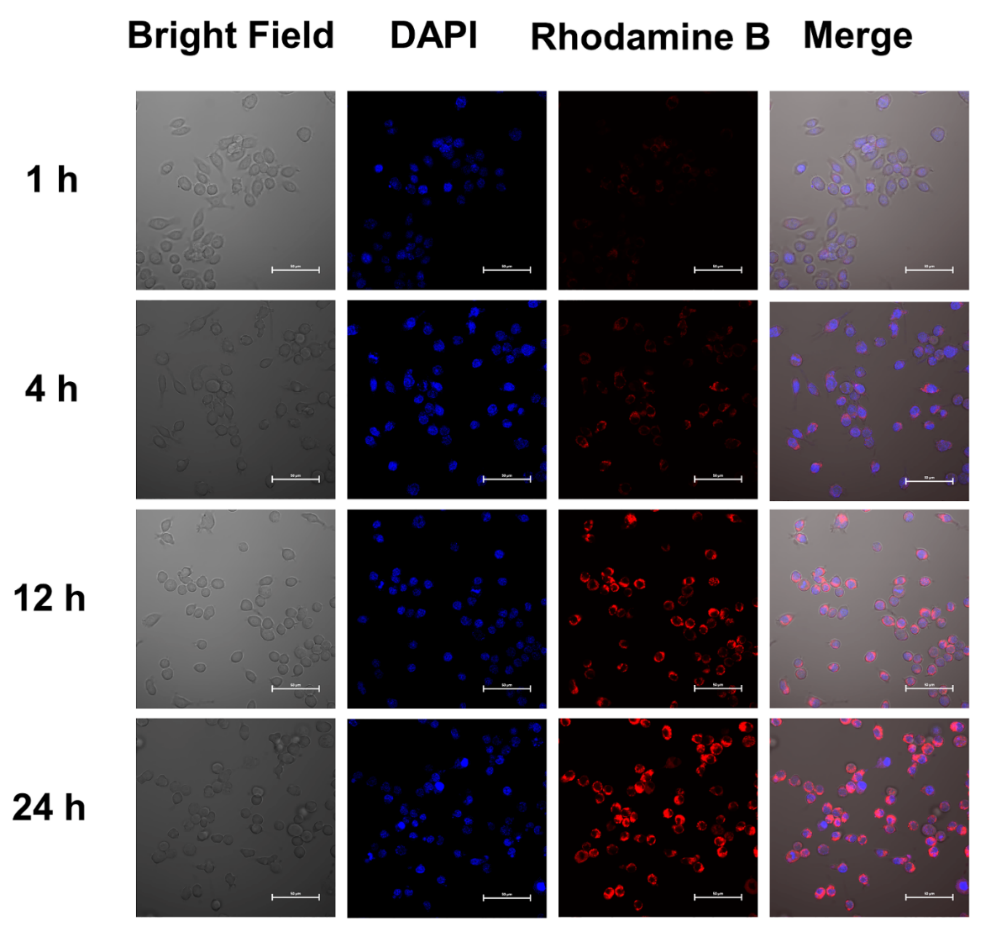


**Figure S16.** CLSM images of RAW264.7 cells incubated with rhodamine-labelled RuFOMs-FA for 1, 4, 12, 24 h. Scale bar: 50 μm.


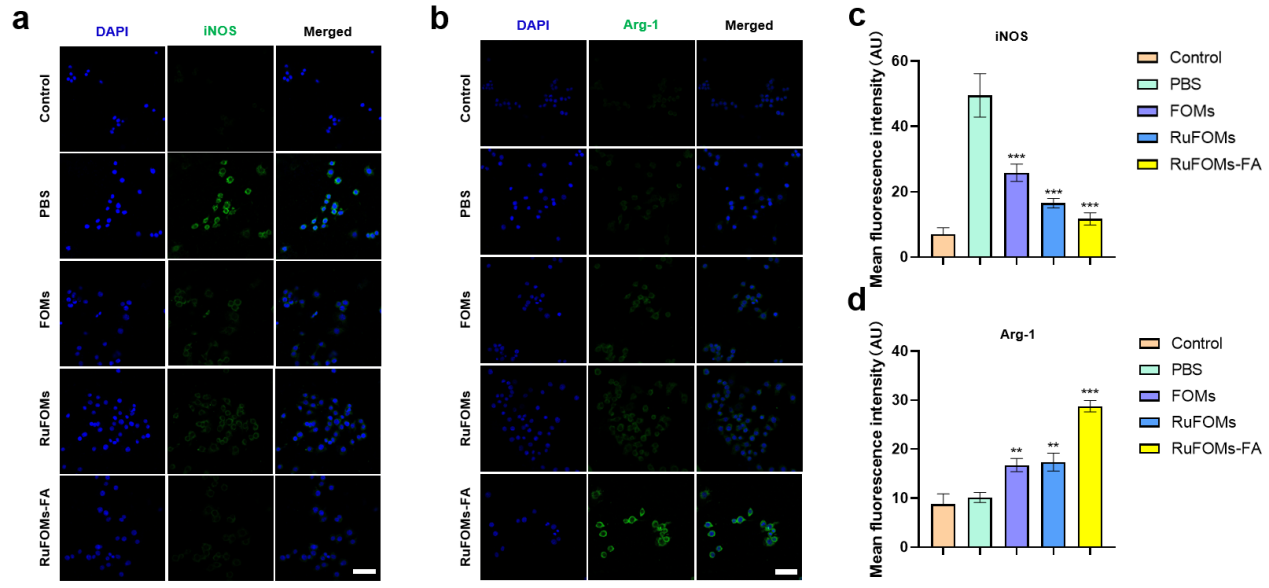


**Figure S17.** Pro-inflammatory M1 to anti-inflammatory M2 phenotypic transition of macrophages induced by NPs *in vitro*. (a) pro-inflammatory M1 biomarker (iNOS; green), (b) anti-inflammatory M2 biomarker (Arg-1; green) and (c-d) the relative quantification in RAW264.7 under various conditions. * indicates significant differences compared with the PBS groups (p < 0.05). **0.001< p < 0.01 vs. PBS group; ***p < 0.001 vs. PBS group. n = 3. Scale bar = 50 μm.


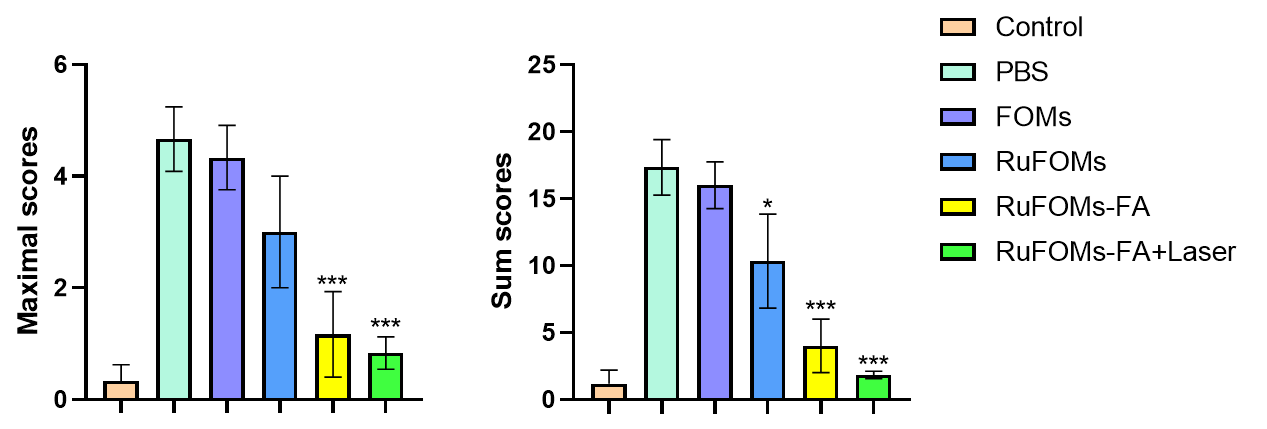
 **Figure S18.** The relative quantification of OA progression by treating various groups was evaluated by the Osteoarthritis Research Society International (OARSI) scoring system. * indicates significant differences compared with the PBS groups (p < 0.05). **0.001< p < 0.01 vs. PBS group; ***p < 0.001 vs. PBS group.


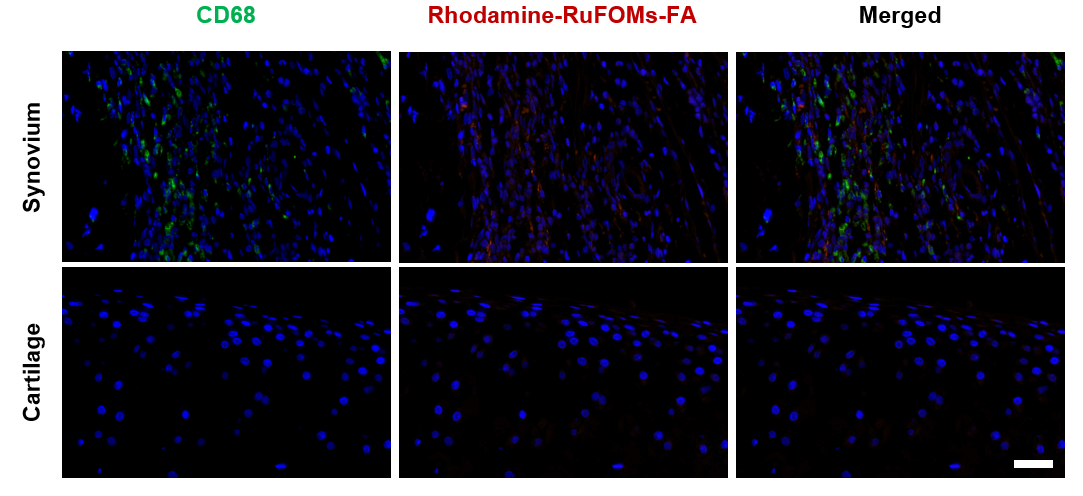


**Figure S19.** Immunofluorescence staining images indicating the internalization of Rhodamine-RuFOMs-FA by synovial macrophages, CD68 antibody (green) was used for staining macrophages, DAPI (blue was used for visualization of nucleus). Scale bar = 25 μm.

**Table S1.** The photothermal conversion efficiency reported from different references.

| Materials | η | Wavelength (nm) | Power density (W cm^-2^) | Refs |
| --- | --- | --- | --- | --- |
| Au@CeO_2_ | 33.7% | 808 | 1 | [5] |
| PEG-dBSA-RuS_1.7_ NCs | 28.5% | 808 | 1.4 | [6] |
| HA-Ru NAs | 37.4% | 808 | 1 | [7] |
| QCS-RBT@RuO_2_ NSs | 53.0% | 808 | 1 | [8] |
| PVP-RuO_2_ | 54.8% | 808 | 1 | [9] |
| RuO_2_@BSA@IR-808-Br_2_ | 33.6% | 808 | 1 | [10] |
| Au_2_Pt-PEG-Ce6 | 31.5% | 808 | 1 | [11] |
| Ni_3_S_2_/Cu_1.8_S@HA | 49.5% | 808 | 0.75 | [12] |
| RuNPs | 53.2% | 808 | 2 | [13] |
| RBCm@Ru@MnO_2_ | 36.99% | 808 | 1 | [14] |

**Table S2.** The K_m_ and V_m_ of mimicking CAT from different papers.

| Materials | K_m_ (M) | V_m­_ (mg L^-1^s^-1^) | Reference |
| --- | --- | --- | --- |
| RuO_2_@OVA NAs | 0.32 | \ | [15] |
| RuO_2_-PVP NPs | 0.843 | 10.7 | [16] |
| Multi-caged IrO_x_ NPs | 0.188 | 0.18 | [17] |
| RuO_2_ NPs | 0.4 | K_cat_=4.4*10^5^ s^-1^ | [18] |
| Catalase from A. niger | 0.465 | K_cat_=2.8*10^5^ s^-1^ | [19] |
| Catalase from B. fragilis | 0.279 | K_cat_=3.8*10^5^ s^-1^ | [19] |

**References**

[1] J.Y. Ko, J. Lee, J. Lee, G.I. Im, Intra-articular Xenotransplantation of Adipose-Derived Stromal Cells to Treat Osteoarthritis in a Goat Model, Tissue. Eng. Regen. Med. 14(1) (2017) 65-71.

[2] N. Schmitz, S. Laverty, V.B. Kraus, T. Aigner, Basic methods in histopathology of joint tissues, Osteoarthritis Cartilage 18 Suppl 3(1522-9653 (Electronic)) (2010) S113-6.

[3] K.P. Pritzker, S. Gay, S.A. Jimenez, K. Ostergaard, J.P. Pelletier, P.A. Revell, D. Salter, W.B. van den Berg, Osteoarthritis cartilage histopathology: grading and staging, Osteoarthritis Cartilage 14(1) (2006) 13-29.

[4] P. Chen, C. Xia, S. Mei, J. Wang, Z. Shan, X. Lin, S. Fan, Intra-articular delivery of sinomenium encapsulated by chitosan microspheres and photo-crosslinked GelMA hydrogel ameliorates osteoarthritis by effectively regulating autophagy, Biomaterials 81 (2016) 1-13.

[5] S. Wang, R. Chen, Q. Yu, W. Huang, P. Lai, J. Tang, L. Nie, Near-infrared plasmon-boosted heat/oxygen enrichment for reversing rheumatoid arthritis with metal/semiconductor composites, ACS Appl. Mater. Inter. 12(41) (2020) 45796-45806.

[6] X. Zhu, X. Chen, Z. Jia, D. Huo, Y. Liu, J. Liu, Cationic chitosan@ruthenium dioxide hybrid nanozymes for photothermal therapy enhancing ROS-mediated eradicating multidrug resistant bacterial infection, J. Colloid. Inter. Sci. 603 (2021) 615-632.

[7] Z. Lu, F.Y. Huang, R. Cao, L. Zhang, G.H. Tan, N. He, J. Huang, G. Wang, Z. Zhang, Long blood residence and large tumor uptake of ruthenium sulfide nanoclusters for highly efficient cancer photothermal therapy, Sci. Rep. 7 (2017) 41571.

[8] W.L. Wang, Z. Guo, Y. Lu, X.C. Shen, T. Chen, R.T. Huang, B. Zhou, C. Wen, H. Liang, B.P. Jiang, Receptor-mediated and tumor-microenvironment combination-responsive Ru nanoaggregates for enhanced cancer phototheranostics, ACS Appl. Mate. Inter. 11(19) (2019) 17294-17305.

[9] Z. Xiao, X. Jiang, B. Li, X. Liu, X. Huang, Y. Zhang, Q. Ren, J. Luo, Z. Qin, J. Hu, Hydrous RuO_2_ nanoparticles as an efficient NIR-light induced photothermal agent for ablation of cancer cells in vitro and in vivo, Nanoscale 7(28) (2015) 11962-70.

[10] P. Xu, X. Wang, T. Li, H. Wu, L. Li, Z. Chen, L. Zhang, Z. Guo, Q. Chen, Biomineralization-inspired nanozyme for single-wavelength laser activated photothermal-photodynamic synergistic treatment against hypoxic tumors, Nanoscale 12(6) (2020) 4051-4060.

[11] M. Wang, M. Chang, Q. Chen, D. Wang, C. Li, Z. Hou, J. Lin, D. Jin, B. Xing, Au_2_Pt-PEG-Ce_6_ nanoformulation with dual nanozyme activities for synergistic chemodynamic therapy/phototherapy, Biomaterials 252 (2020) 120093.

[12] D. Sang, K. Wang, X. Sun, Y. Wang, H. Lin, R. Jia, F. Qu, NIR-driven intracellular photocatalytic O_2_ evolution on Z-scheme Ni_3_S_2_/Cu_1.8_S@HA for hypoxic tumor therapy, ACS Appl. Mater. Inter. 13(8) (2021) 9604-9619.

[13] S. Zhao, X. Zhu, C. Cao, J. Sun, J. Liu, Transferrin modified ruthenium nanoparticles with good biocompatibility for photothermal tumor therapy, J Colloid. Interf. Sci 511 (2018) 325-334.

[14] X. Zhu, X. Chen, D. Huo, J. Cen, Z. Jia, Y. Liu, J. Liu, A hybrid nanozymes in situ oxygen supply synergistic photothermal/chemotherapy of cancer management, Biomater. Sci. 9(15) (2021) 5330-5343.

[15] R. Huang, Z. Ding, B.P. Jiang, Z. Luo, T. Chen, Z. Guo, S.C. Ji, H. Liang, X.C. Shen, Artificial metalloprotein nanoanalogues: In situ catalytic production of oxygen to enhance photoimmunotherapeutic inhibition of primary and abscopal tumor growth, Small 16(46) (2020) 2004345.

[16] W. C, H. X, F. W, Z. Liu, L. Chen, B. Zhou, Y. Chen, J. Shi, Multi-enzymatic activities of ultrasmall ruthenium oxide for anti-inflammation and neuroprotection, Chem. Eng. J. 411(1) (2021) 128543-128556.

[17] W. Zhen, Y. Liu, W. Wang, M. Zhang, W. Hu, X. Jia, C. Wang, X. Jiang, Specific "unlocking" of a nanozyme-based butterfly effect to break the evolutionary fitness of chaotic tumors, Angew Chem Int Ed Engl 59(24) (2020) 9491-9497.

[18] H. Deng, W. Shen, Y. Peng, X. Chen, G. Yi, Z. Gao, Nanoparticulate peroxidase/catalase mimetic and its application, Chemistry (Easton) 18(29) (2012) 8906-11.

[19] P.C.L. Jacek Switala, Diversity of properties among catalases, Arch. Biochem. Biophys. 401(2) (2002) 145-154.
